# Precise exogenous insertion and sequence replacements in poplar by simultaneous HDR overexpression and NHEJ suppression using CRISPR-Cas9

**DOI:** 10.1093/hr/uhac154

**Published:** 2022-07-22

**Authors:** Ali Movahedi, Hui Wei, Xiaohong Zhou, Jake C Fountain, Zhong-Hua Chen, Zhiying Mu, Weibo Sun, Jiaxin Zhang, Dawei Li, Baozhu Guo, Rajeev K Varshney, Liming Yang, Qiang Zhuge

**Affiliations:** College of Biology and the Environment, Co-Innovation Center for Sustainable Forestry in Southern China, Key Laboratory of Forest Genetics & Biotechnology, Ministry of Education, Nanjing Forestry University, Nanjing 210037, China; College of Biology and the Environment, Co-Innovation Center for Sustainable Forestry in Southern China, Key Laboratory of Forest Genetics & Biotechnology, Ministry of Education, Nanjing Forestry University, Nanjing 210037, China; College of Forestry and Biotechnology, Zhejiang Agriculture and Forestry University, Hangzhou 311300, China; Department of Biochemistry, Molecular Biology, Entomology, and Plant Pathology, Mississippi State University, Mississippi State, MS 39762, USA; School of Science, Hawkesbury Institute for the Environment, Western Sydney University, Penrith, NSW 2751, Australia; College of Forestry and Biotechnology, Zhejiang Agriculture and Forestry University, Hangzhou 311300, China; College of Biology and the Environment, Co-Innovation Center for Sustainable Forestry in Southern China, Key Laboratory of Forest Genetics & Biotechnology, Ministry of Education, Nanjing Forestry University, Nanjing 210037, China; School of Food Science and Pharmaceutical Engineering, Nanjing Normal University, Nanjing 210046, China; College of Biology and the Environment, Co-Innovation Center for Sustainable Forestry in Southern China, Key Laboratory of Forest Genetics & Biotechnology, Ministry of Education, Nanjing Forestry University, Nanjing 210037, China; USDA-ARS, Crop Genetics and Breeding Research Unit, Tifton, GA 31793, USA; Center of Excellence in Genomics & Systems Biology, International Crops Research Institute for the Semi-Arid Tropics (ICRISAT), Hyderabad 502324, India; State Agricultural Biotechnology Center, Center for Crop and Food Innovation, Food Futures Institute, Murdoch University, Murdoch, Western Australia 6150, Australia; College of Biology and the Environment, Co-Innovation Center for Sustainable Forestry in Southern China, Key Laboratory of Forest Genetics & Biotechnology, Ministry of Education, Nanjing Forestry University, Nanjing 210037, China; College of Biology and the Environment, Co-Innovation Center for Sustainable Forestry in Southern China, Key Laboratory of Forest Genetics & Biotechnology, Ministry of Education, Nanjing Forestry University, Nanjing 210037, China

## Abstract

CRISPR-mediated genome editing has become a powerful tool for the genetic modification of biological traits. However, developing an efficient, site-specific, gene knock-in system based on homology-directed DNA repair (HDR) remains a significant challenge in plants, especially in woody species like poplar. Here, we show that simultaneous inhibition of non-homologous end joining (NHEJ) recombination cofactor XRCC4 and overexpression of HDR enhancer factors CtIP and MRE11 can improve HDR efficiency for gene knock-in. Using this approach, the *BleoR* gene was integrated onto the 3′ end of the *MKK2* MAP kinase gene to generate a BleoR-MKK2 fusion protein. Based on fully edited nucleotides evaluated by TaqMan real-time PCR, the HDR-mediated knock-in efficiency was up to 48% when using *XRCC4* silencing incorporated with a combination of *CtIP* and *MRE11* overexpression compared with no HDR enhancement or NHEJ silencing. Furthermore, this combination of HDR enhancer overexpression and NHEJ repression also increased genome targeting efficiency and gave 7-fold fewer CRISPR-induced insertions and deletions (InDels), resulting in no functional effects on *MKK2*-based salt stress responses in poplar. Therefore, this approach may be useful not only in poplar and plants or crops but also in mammals for improving CRISPR-mediated gene knock-in efficiency.

## Introduction

Several studies have been carried out on improving crop genetic modification by CRISPR-mediated donor-dependent homology-directed DNA repair (HDR) [1], such as increasing *ARGOS8* expression by replacing the *GOS2* promoter with HDR and enhancing the efficiency of 35S promoter insertion upstream of the *ANT1* gene in tomato [2]. Several publications have reported the successful generation of null mutations in woody plants using the non-homologous end joining (NHEJ) pathway since it was implemented in poplar [3, 4]. However, precise gene targeting and replacement have only been reported in model plants, such as *Arabidopsis* [5] and rice [6]. No report has yet shown efficient HDR for gene replacement in woody perennials.

One of the main limitations of HDR efficiency is inadequate delivery of donor DNA patterns (DDPs) into nuclei. Previous studies have indicated that it is necessary to increase the number of cells containing DDPs at S and G2 cell division phases to increase HDR efficiency [7]. Several traditional strategies have been applied to increase DDP availability and introduction into cells, including particle bombardment [8], protoplasts [9], geminiviral-based replication [10], and RNA transcription [1], but it remains a significant problem for woody plants. Although genes introduced by *Agrobacterium* are stable and the method is widely used to transduce genes into woody plant cells [11, 12], there have been few reports on enhancing the efficiency of transferring DDPs and, consequently, the recovery of double-strand breaks (DSBs) by HDR [13, 14].

Cas9 integrates with MRE11, CtIP, Rad51, and Rad52, promoting meaningful increases in HDR efficiency in human cells while significantly decreasing NHEJ with at least a 2-fold increase in HDR and a 6-fold increase in HDR/NHEJ ratio [15]. On the other hand, inhibition of DNA ligase IV (LIG4), Ku 70, and Ku 80, which are outwardly involved only in NHEJ and known as the most critical NHEJ factors, protect DSBs by forming one heterodimeric complex to bind tightly and load additional repair proteins such as DNA ligase IV [15–19]. This inhibition has also been shown to increase HDR efficiency up to 19-fold [15]. In *Arabidopsis*, mutated *Ku70* or *Lig4* enhanced HDR-based genome targeting 16-fold or 4-fold, respectively [20].

Here we applied X-ray repair cross-complementing protein 4 (XRCC4), another critical NHEJ factor that has not yet been considered for its interfering effect on HDR efficiency. XRCC4 is a cofactor of LIG4 that interacts with Ku 70 and Ku 80 and ligates the DSB [21, 22]. XRCC4 has already been demonstrated to be an NHEJ potent effector in DNA repair and to affect HDR efficiency in mammals [21]. Loss of function of this protein induces HDR efficiency enhancement [18]. However, it has not been widely considered for its interfering effect on HDR efficiency in plants, and this study could be considered the first to investigate the effects of *XRCC4* mutation on HDR efficiency in plants.

To date, there has been no report of combining HDR factor overexpression and NHEJ factor suppression to promote HDR efficiency in plants. Intense gene targeting and knock-in by homologous recombination are more challenging but necessary as a versatile tool for research and breeding in crops and woody plants. Therefore, our objective was to examine the effects of HDR cofactor overexpression (*CtIP* and *MRE11*) [15] and simultaneous disruption of the NHEJ promoter *XRCC4* [18] on knock-in efficiency using the *MKK2* gene as a case study. Mitogen-activated protein kinases (MAPKs or MPKs) like *MKK2* are involved in several key pathways responding to stresses, including disease, drought, cold, heat shock, osmotic, and salt stresses [23, 24].

## Results

### CRISPR/Cas9 edited *MKK2* based on efficient HDR

**Figure 1:**
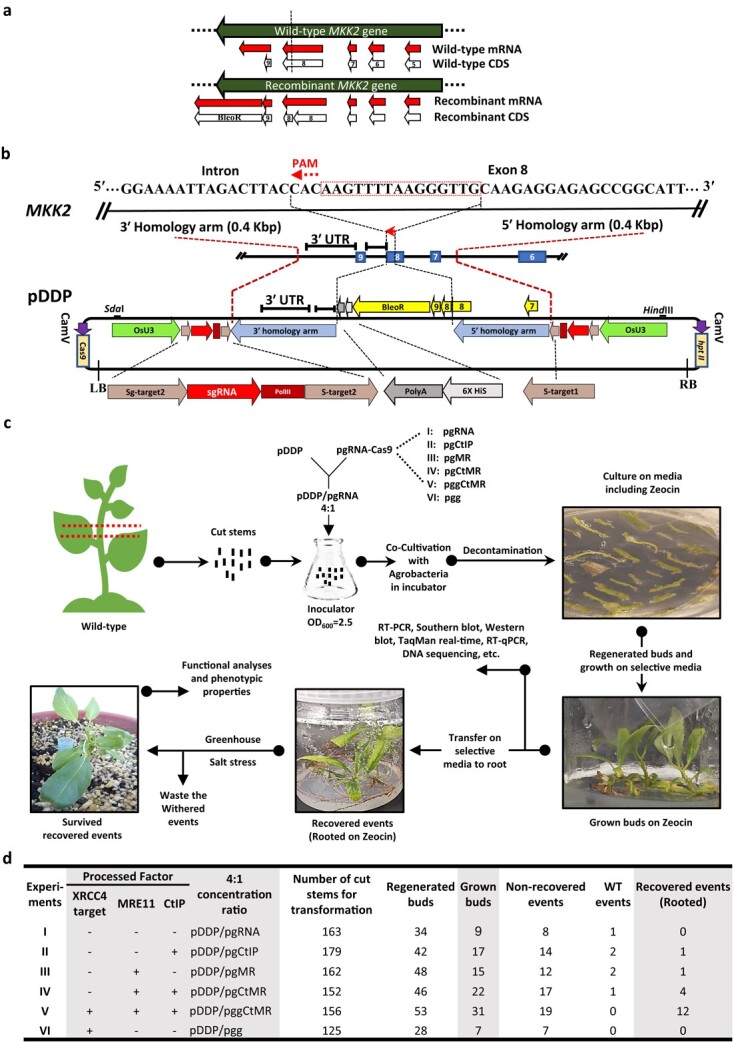
Exogenous *BleoR* CDS is integrated into the poplar genome. **a** The purpose of this study was to generate recombinant mRNA, including *MKK2* and *BleoR*. The dashed line reveals the target site. **b** The protospacer-adjacent motif (PAM) was detected at exon 8 to lead Cas9. 400 bp sequences from up- and downstream of the CRISPR target were picked for HDR in this study. The 5′ homology arm included part sequences of the intron between exon 6 and 7, exon 7, intron sequences between exons 7 and 8, and a part of exon 8. The 3′ homology arm included intron sequences between exons 8 and 9 and the 3′-UTR of the *MKK2* locus up to 400 bp. The designed DDP included remaining sequences of exon 8, exon 9, *BleoR* CDS, 6xHis, and Poly-A sequences flanked by the 3′ and 5′ homology arms. In addition, two special targets (S-target1 and 2) (with no on-and-off-targets through the whole poplar genome) were designed to attach both sides of the DDP. The DDP was then ligated into the pRGEB31 vector to form pDDP. **c**, pDDP and pgRNA-Cas9 were mixed 4:1 and introduced into *A. tumefaciens* to form an inoculator suspension and condensed to OD_600_ = 2.5. The putatively edited events were regenerated on Zeocin and were allowed to bud. The grown buds were then transferred on selective rooting media until they could be recovered. Recovered events were then planted on soil, followed by salt stress. **d** Overview of designed experiments with the numbers of recovered (Rooted) events, including (I) no HDR factors, (II) overexpressed *CtIP*, (III) overexpressed *MRE11*, (IV) overexpressed *CtIP + MRE11*, (V) overexpressed *CtIP + MRE11* with *XRCC4* deficiency, and (VI) *XRCC4* deficiency without overexpressed *CtIP + MRE11.*

Regarding highly efficient CRISPR-mediated homozygous mutations in poplars [25], the *MKK2* gene was targeted to integrate the Zeocin resistance gene *BleoR* via designed guide RNA (gRNA) near the 3′ UTR with the highest activity score and no off-target effects on the coding sequences (CDS) to avoid impact on expression and function (Fig. 1a; Supplementary Data Table 1) [26, 27]. According to Song *et al*. [28], homologous arm lengths were optimized for homologous recombination and *BleoR* integrations to 400 bp upstream and downstream of the protospacer-adjacent motif (PAM) site were designated as the 5′ and 3′ homology arms, respectively (Fig. 1b). The DDP cassette was ligated into the pRGEB31 vector containing the Cas9 expression cassette to construct the pDDP vector (Supplementary Data Fig. S1). Multiple fusion vectors were also constructed to manipulate HDR and NHEJ cofactors, including the Cas9 expression cassette, *CtIP* overexpression cassette, *MRE11* overexpression cassette, and *XRCC4* mutative cassettes (Supplementary Data Fig. S2a–f). We used the pathogenic suspension of *Agrobacterium tumefaciens* with OD_600_ = 2.5 (~2 × 10^9^ cells ml^−1^) and the ratio of 4:1 pDDP/pgRNA (Fig. 1c). Actively growing buds on Zeocin-containing selection medium were assumed to be positive transformants and were selected and subcultured on Zeocin-containing selection medium to root (recovery). The recovered events were then used for further analyses (Fig. 1c).

The pDDP cassette was co-transformed with one of five additional constructs in a 4:1 concentration ratio of pgRNA (Experiment I; ExI), pgCtIP overexpressing HDR effector CtIP (Experiment II; ExII), pgMR overexpressing HDR effector MRE11 (Experiment III; ExIII), pgCtMR overexpressing both CtIP and MRE11 (Experiment IV; ExIV), pggCtMR overexpressing both *CtIP* and *MRE11* along with CRISPR-based disruption of NHEJ effector *XRCC4* (Experiment V; ExV), and pgg with CRISPR-based disruption of NHEJ effector *XRCC4* (Experiment VI; ExVI) to examine the effect of increasing HDR effector expression and disruption of the NHEJ component on knock-in efficiency. While ExI resulted in no successfully recovered lines being generated from nine grown buds, ExII and ExIII resulted in one recovered event from 17 and 15 grown buds, respectively (Fig. 1d). Combining *CtIP* and *MRE11* overexpression with pggCtMR resulted in four recovered events from 22 grown buds in ExIV while combining overexpression of *CtIP* and *MRE11* with the disruption of *XRCC4* resulted in a significant increase in recovered events to 12 from 31 grown buds (Fig. 1d). Disrupting NHEJ effector *XRCC4* with no *CtIP* and *MRE11* overexpression support resulted in no recovered events from seven grown buds in ExVI. These recovered events in ExV let us assume that CRISPR-based disruption of *XRCC4* linked by *CtIP* and *MRE11* overexpression leads to increased knock-in efficiency of the *BleoR* gene.

### Transformants verified via western blotting, RT–PCR, and Southern blotting

**Figure 2:**
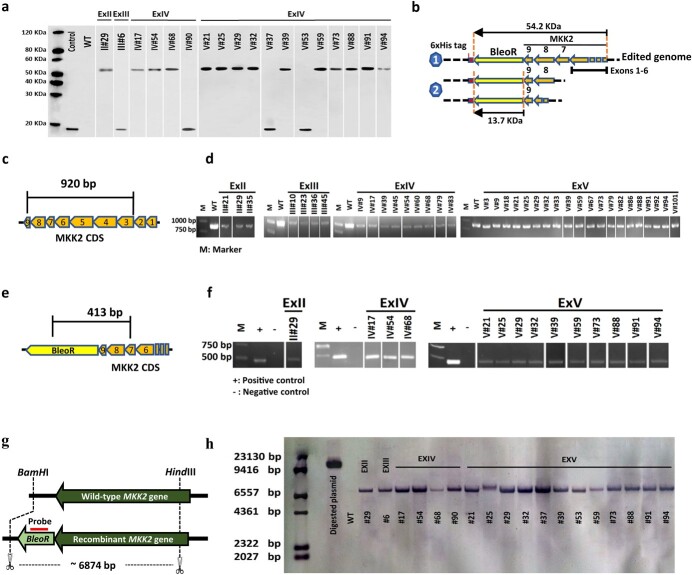
Western blotting, RT–PCR, and Southern blotting exhibited integrated *BleoR* expression in recovered events. **a** Recovered events exhibited BleoR expression by western blotting. **b** Schematic of fusion 6xHis tag with edited poplar genome triggered by different constructions. Shape 1 reveals successful fusion of *BleoR* and MKK2 with ~54.2 kDa. Shape 2 reveals only BleoR translation and unsuccessful fusion of MKK2 and BleoR proteins because of MKK2 disruption with ~13.7 kDa. **c** Proper HDR resulted in attachment of exons 8 and 9 resulting in MKK2 transcription in the edited genome. **d** RT–PCR showed the actual engineered events resulting in amplifying 920 bp of MKK2 CDS. β-Actin was used in all RT–PCR assays for normalization; WT was used as the positive control. **e** Proper integration in the edited genome caused integration of BleoR in the C-terminal of MKK2. **f** RT–PCR detected the recovered events with proper integration of BleoR and MKK2 with amplification of 413 bp of the transcribed sequence. β-Actin was used in all RT–PCR assays for normalization; pDDP plasmid was used as the positive control. WT was used as the negative control. **g** Schematic of probing *BleoR* in edited events and WT as the control using Southern blotting. **h** Southern blotting proved the integration of *BleoR* into the poplar genome, consistent with the western blotting results. Linearized pDDP plasmid was used as the positive control; WT was used as the negative control.

Western blotting, RT–PCR, and Southern blotting were used to verify that HDR occurred in recovered transformants and to confirm the proper integration of *BleoR* in the 3′ end of the *MKK2* gene. A 6XHis tag was fused with the BleoR C-terminal (Fig. 1b), followed by a Poly-A tail to show the integration of *BleoR* into target genomes using western blotting. While screening the transformants grown on Zeocin using western blotting, no edited events were detected in ExI, but one event showed a band of 54 kDa in ExII (Fig. 2a; uncropped images in Supplementary Data Fig. S3), which represent the successful integration of BleoR (~13.7 kDa) fused with MKK2 (~40.5 kDa) (Fig. 2b). In screening events in ExIII, only one with a band of 13.7 kDa (Fig. 2a) was identified, suggesting that the *BleoR* CDS was integrated but did not successfully form a fusion protein with MKK2, possibly due to mutation or knock-out of *MKK2* exon 7, 8, or 9 (Fig. 2b). Screening events in ExIV showed three events with ~54-kDa bands and one event with ~14 kDa (Fig. 2a). Simultaneous HDR effector overexpression and NHEJ suppression in ExV resulted in 10 events with ~54-kDa bands and two events with ~14-kDa bands (Fig. 2a). However, NHEJ suppression in ExVI resulted in no significant bands appearing in western blotting.

The proper arrangement of nucleotides within HDR causes transcription of the allocated genes. Therefore, we performed RT–PCR to verify the precise attachment of exons 8 and 9 to direct the transcription of *MKK2* correctly. In addition, the proper orientation of inserted *BleoR* at the 3′ end of exon 8 caused transcription driven by the *MKK2* genomic promoter. The first RT–PCR experiment was designed to determine whether HDR was successful and proper *MKK2* gene transcription occurred in the transformants (Fig. 2c and d). With wild-type (WT) transcription bands as the control, we amplified a 920-bp region spanning exons 3–9. No bands were observed in ExI and ExVI events, while three ExII-, four ExIII-, and nine ExIV events showed 920-bp bands. The second RT–PCR experiment evaluated the appropriate orientation of inserted *BleoR* at the 3′ end of *MKK2*, in which pDDP was designed as the positive control. The 413-bp amplicon was used to prove the transcription of *BleoR* happened by proper knock-in HDR (Fig. 2e and f). While no band was observed in ExI-, ExIII-, and ExVI events (which indicated no proper *BleoR* integration), ExII events showed only one band. In addition, ExIV and ExV showed 3 and 10 positive bands, respectively (Fig. 2f).

To validate *leoR* knock-in, Southern blotting was performed using a *BleoR*-specific probe for all recovered events (Fig. 2g and h). A single band for each event proved the precise integration in the desired positions with one or more copy numbers, indicating the lack of *BleoR* knock-in elsewhere in the off-target sites. While the ExI- and ExII events revealed no detected probe, more achieved bands were observed in ExV events than in other experiments. Overall, comparing ExV results with the other experiments further supports the concept that the overexpression of *CtIP* and *MER11* and disruption of *XRCC4* led to increased knock-in efficiency.

### *MKK2* and *XRCC4* targeting efficiency verified by T7E1 and genotyping

*MKK2* genome targeting efficiency was assessed by T7 endonuclease I (T7EI) (Supplementary Data Fig. S4a and b). Comparing on-target and CRISPR/Cas9-influenced mismatches occurring in all recovered events revealed that *XRCC4* deficiency combined with *CtIP* and *MRE11* overexpressions (ExV) caused T7EI mismatch cleavage band numbers and densities to be less than in the other experiments, improving on-target efficiency to the optimized ~74.89% (#V88) (Supplementary Fig. S4a and b). Mismatch nucleotide cleavage analyses revealed most transformants as heterozygous knock-ins compared with the homozygous *MKK2* gene in treated WT and untreated poplars.

T7EI was also applied to evaluate genome targeting efficiency and determine the rate of on-target efficiency in ExV events via the designed *XRCC4* targeting (Supplementary Data Fig. S4c and d). In total, 19.35% of ExV events revealed ~100% on-target efficiency, and 54.83% of them indicated ~50% on-target efficiency. These results further revealed the homozygosity of the *XRCC4* gene in WT poplars and several recovered transformants in ExV (#21, #25, #29, #88, #91, and #94) compared with heterozygous mutations among knocked-in poplars*.* The on-target genotyping of *XRCC4* resulting from the T7E1 assay confirmed the knock-out of *XRCC4* with the bulk of InDels that happened through the ExV events genome (Supplementary Data Fig. S6a). The off-target genotyping affected by *XRCC4* targeting showed only one was off-target with no impact on *XRCC4* expression via ExV events (Supplementary Data Fig. Sb).

T7EI was further applied to evaluate on-target efficiency through *XRCC4* targeting in ExVI events. These results revealed >55% on-target efficiency in all ExVI events (Supplementary Data Fig. S6a and b). These results also proved the homozygosity of the *XRCC4* gene in WT poplars and several recovered transformants compared with heterozygous mutations among knocked-in poplars*.* The same genotyping analyses in *XRCC4* targeting through all ExVI events proved the appropriate on-target efficiency to knock out *XRCC4*, generating proper mutant poplars with a large number of happend InDels (Supplementary Data Fig. S6c). The off-target genotyping affected by *XRCC4* targeting exhibited the same results with the ExV events (Supplementary Data Fig. S5b).

### Accurate HDR efficiency was achieved by *XRCC4* knock-out

**Figure 3:**
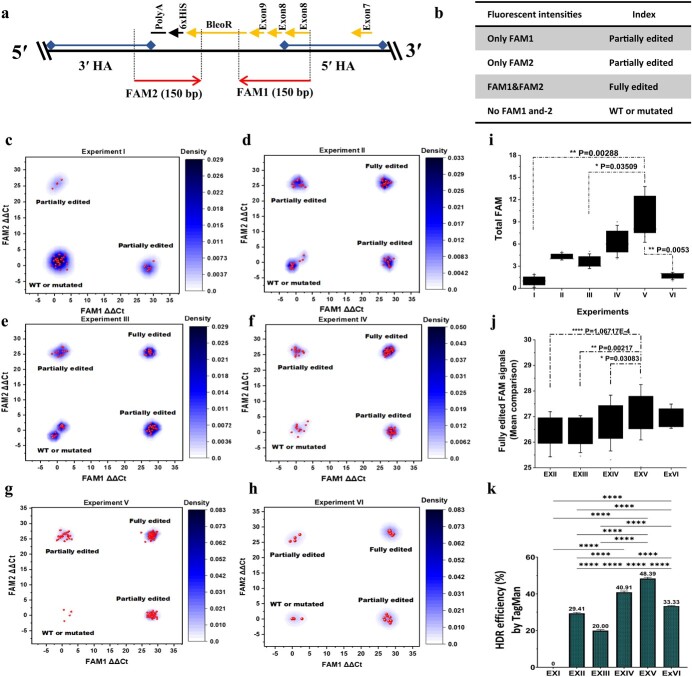
TaqMan real-time PCR to validate and evaluate HDR occurrence and efficiency. **a** Designing TaqMan real-time PCR assay to detect and evaluate HDR efficiency, including FAM1 and FAM2 DNA binding probes. **b** Strategy to classify edited events. **c** Experiment I revealed no edited events. **d** The density plot of FAM1 and 2 intensities resulting from experiment II revealed expansion of edited events against partial, mutant, and WTs. **e** The density plot of FAM1 and 2 signals resulting from experiment III revealed an increased intensity of partial FAM1 events. **f** Experiment IV revealed a marked increase in edited event signals in comparison with three earlier experiments. **g** Density plot of experiment V revealed a significant increase in FAM1 and 2 intensities in edited events compared with the earlier experiments and a significant decrease in intensities in WT and mutated events. **h** Experiment VI exhibited more partial than fully edited FAM signals. All samples were analyzed in quadruplicate. **i** Diamond box and whisker plot comparison of FAM signals (partial FAM1 and 2, and FAM1 and 2) detected in all experiments, showing markedly more signals measured in ExV than ExI, III, and VI; error bars represent standard error; ^*^P ≤ .05, ^**^P ≤ .01. **j** Fully edited FAM signals (ΔΔCt) from each experiment were evaluated for a comparison of means; error bars represent standard error; ^*^P ≤ .05, ^**^P ≤ .01, ^****^P ≤ .0001. **k** HDR efficiency (%) was calculated based on fully edited replicate numbers for each experiment. ExV events revealed significantly greater HDR efficiency (%) than other events, ~48%. Error bars represent standard deviation; ^****^P ≤ .0001.

TaqMan real-time PCR was utilized to evaluate HDR efficiency using probes FAM1 (which included the 5′ ends of the *BleoR* CDS and exons 8 and 9 of *MKK2*) and FAM2 (which included the 3′ end of the *BleoR* CDS plus a few nucleotides of the 3′ homology arm) (Fig. 3a) and validated by Sanger sequencing, followed by multisequence alignment. Transformants exhibiting both FAM1 and FAM2 fluorescent signals were assumed to be fully edited (Fig. 3b). Those showing only FAM1 or FAM2 were considered to be partially edited, and those with no FAM1 or FAM2 signals were supposed to be either mutants or WT (Fig. 3b). In ExI, the averages of fluorescent signal numbers of FAM1 and FAM2 ∆∆Ct were between 8 and 4 (Supplementary Data Fig. S7a), and most events appeared as mutant or WT, with a few having partial FAM1 or FAM2 fluorescence (Fig. 3c; Supplementary Data Fig. S8). The ExII and ExIII events showed enhanced fully and partially edited FAM signal numbers (Fig. 3d and e; Supplementary Data Fig. S7b and c). In ExII, there were four fully edited events, four FAM1 partially edited events, and four FAM2 partially edited events (Supplementary Data Figs S9 and S14a). In ExIII, three fully edited events, five FAM1 partially edited events, and three FAM2 partially edited events were observed (Supplementary Data Figs S10 and S14b). In ExIV, the signal density of edited events increased significantly (Fig. 3f). The mean fluorescent FAM1 and FAM2 signal numbers showed an increase of ~20 and 15, respectively (Supplementary Data Fig. S7d). In total, nine fully edited events, seven FAM1 partially edited events, and four FAM2 partially edited events (Supplementary Data Figs S11 and S14c) were detected. The FAM signal densities of fully edited transformants in ExV were increased (Fig. 3g) with means of ~21 and 19, respectively (Supplementary Data Fig. S7e), and 15 fully edited events were discovered (Supplementary Data Figs S12 and S14d). Finally, ExVI events revealed two fully FAM1 and FAM2 edited events and four partially FAM1 or FAM2 edited events (Fig. 3h) with means of ~14 and 14, respectively (Supplementary Data Figs S13 and S14e). Furthermore, total FAM fluorescent signals (FAM1, FAM2, and FAM1 + 2) indicated a significant increasing HDR occurrence trend through ExV events compared with the other experiments (Fig. 3i). These results revealed that *XRCC4* silencing combined with *CtIP* and *MRE11* caused a significant increase in achieving the mean of the fully edited FAM signals (ΔΔCt) from ExV compared with the other experiments (Fig. 3j; Supplementary Data Table S3a).

Encouraged by this result, we calculated the HDR efficiency (%) (Fig. 3k; Supplementary Data Tables S3b and c). Our results showed that HDR efficiency significantly increased from 0 in ExI to 29.41 and 20% in ExII (CtIP-Cas9) and ExIII (MRE11-Cas9) events. When fusing both CtIP and MRE11 with Cas9, the HDR efficiency in ExIV meaningfully improved to 40.91%. Our strategy of *XRCC4* deficiency in conjunction with *CtIP* and *MRE11* overexpression (ExV) surprisingly directed HDR by 48.39%. Finally, the only *XRCC4* deficiency (ExVI) exhibited HDR efficiency by 33.33%, significantly more than ExI, II, and III, but meaningfully less than ExIV and V. Overall, *XRCC4* deficiency, supported by the overexpression of *CtIP* and *MRE11*, was the most efficient system for HDR-based integration, resulting in more HDR occurrence than the expression effectors CtIP and MRE11 alone or together or even only NHEJ deficiency (Fig. 3k).

### *XRCC4*, *CtIP,* and *MRE11* expressions verified achievement of efficient HDR

To verify HDR efficiency, we carried out real-time PCR, analyzing and comparing *CtIP*, *MRE11* expressions and *XRCC4* deficiency using all experimental grown bud events (Supplementary Data Fig. S15). Analyses of *CtIP*, *MRE11*, and *XRCC4* expressions and their dependency throughout all experiments showed that in normal conditions NHEJ is a dominant DNA repair system regarding more expression of NHEJ-related genes such as *XRCC4* (Supplementary Data Fig. S15a). The overexpression of HDR-related genes such as *CtIP* or *MRE11* separately caused the control of the expression of XRCC4 in the ExII and III events, leading to an enhanced DNA repair system in the HDR pathway (Supplementary Data Fig. S15b and c). The HDR pathway was then enhanced more by overexpressing *CtIP* and *MRE11* together in ExIV events but could not control the expression of NHEJ-related gene *XRCC4* significantly (Supplementary Data Fig. S15d). In ExV events, the HDR pathway was significantly enhanced among overexpressed *CtIP* and *MRE11*, and deficient *XRCC4* simultaneously caused an increase in fully edited FAM signals and recovered events (Supplementary Data Fig. S15e). In further experiments, only *XRCC4* deficiency raised HDR-related *CtIP* and *MRE11* gene expressions, though not significantly, resulting in a slightly improved HDR DNA repair system with no recovered event (Supplementary Data Fig. S15f). Transformants with no *CtIP* and *MRE11* overexpressions (ExI) revealed more *XRCC4* expression, resulting in no HDR efficiency. The overexpression of *CtIP* (ExII) and *MRE11* (ExIII) could not efficiently overcome the NHEJ pathway significantly and revealed lower HDR efficiency. The simultaneous overexpression of *CtIP* and *MRE11* (ExIV) decreased *XRCC4* expression, resulting in enhancing HDR efficiency. The *XRCC4* deficiency, together with *CtIP* + *MRE11* overexpressions, overcame the NHEJ significantly and improved HDR efficiency (ExV). Finally, the only *XRCC4* deficiency could not lead to significant HDR efficiency (ExVI).

### Dependent expressions of *MKK2* and *BleoR* verified achievement of efficient HDR

Real-time PCR was applied to analyze the expressions of *MKK2* and *BleoR* in response to Zeocin to verify the efficient HDR that occurred in all experimentally grown buds. While the ExI and ExVI events revealed no expressions of *MKK2* and *BleoR* compared with WT poplars, upregulation of *CtIP* and *MRE11* in ExII, ExIII, and ExIV resulted in increasing HDR efficiency with improved quality and quantity of expressions of *MKK2* and *BleoR* (Supplementary Data Fig. S16a). The *XRCC4* deficiency together with upregulated *CtIP* and *MRE11* in ExV events led to increased quality and quantity of expressions of *MKK2* and *BleoR* genes for >110% (Supplementary Data Fig. S16a). In addition, analysis of mean expressions revealed a correlated incremental linear relationship with an increase of 15–59% of *MKK2* and 5–35% of *BleoR* from ExII to ExV events (Supplementary Data Fig. S16b). Dependent expressions of *MKK2* and *BleoR* among the proper fusion of their transcripts verified the accurate and efficient HDR.

### *XRCC4* deficiency dramatically enhanced HDR efficiency and decreased polymorphisms

**Figure 4:**
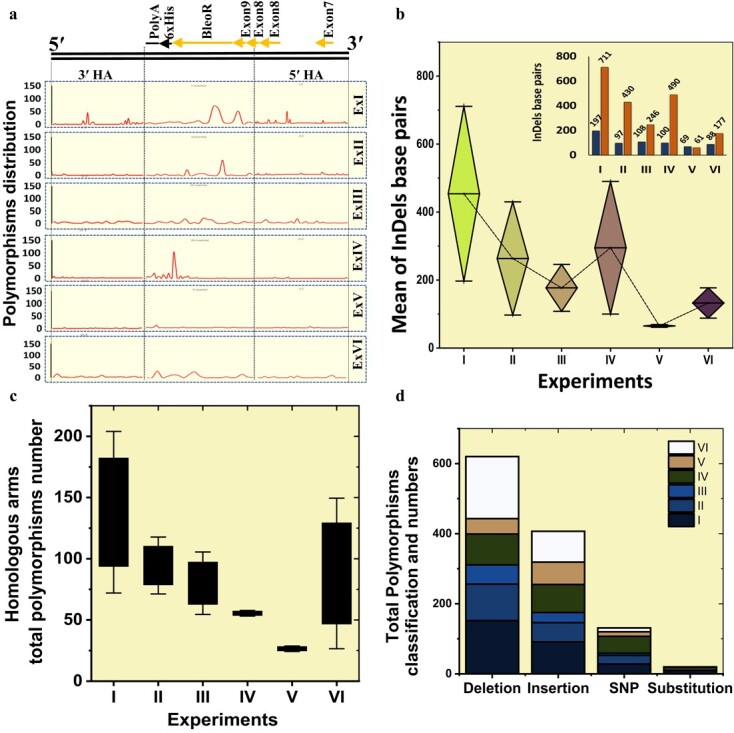
HDR promotion by *XRCC4* deficiency in combination with *CtIP* and *MRE11* overexpression caused a considerable decrease in InDels via ExV events compared with the other experiments. **a** Analysis of distribution of InDels on 5′ and 3′ homology arms (HA) and knocked in fragments throughout experiment events. **b** Diamond box and whiskers for the mean comparisons of total InDel base pairs in all experiment events. The exact numbers of InDels (excluding SNPs and substitutions) are presented via the column bars in the top-right corner. **c** Identification of the polymorphisms found in the homology arms among all the experiments. Box and whisker plot revealed that most polymorphisms happened in homology arms via ExI, and the number was higher than those in ExV and IV. Error bars represent the standard error. **d** Stacked column plot of total polymorphism classification and numbers in DDP integration among all the experiments. Insertions and deletions occurred much more than the other types.

To evaluate the effect of *CtIP* and *MRE11* overexpressions and simultaneous *XRCC4* suppression on CRISPR-induced polymorphisms (insertions, deletions, SNPs, and substitutions), we analyzed detected polymorphisms within the 5′ and 3′ homologous arms and variant genotypes and protein effects of the knocked-in fragments (*MKK2* exons 8 and 9 and *BleoR* CDS) in all the grown buds from each experiment (Supplementary Data Tables S4 and S5). The increased HDR efficiency observed over the following experiments from ExI to ExVI suggested a shift from higher insertion/deletion (InDel) polymorphism occurrence in the 5′ region of knocked-in fragments to the 3′ region (6xHis tag and Poly-A) (Fig. 4a). This shift resulted in less functional disruption of *BleoR* and *MKK2* expressions, resulting in greater quantity and quality of expression in ExV events than in the other experiments. Furthermore, the mean of InDel base pair comparisons revealed that the significant promotion of HDR by *XRCC4* deficiency caused a 7-fold decrease in InDel base pairs through ExV events compared with ExI events and the other experiments (Fig. 4b). We then calculated the total number of polymorphisms throughout the homologous arms in each experiment. ExIV and ExVI events resulted in the fewest polymorphisms, significantly 2.3-fold and 3.8-fold less than in ExI events (Fig. 4c). More polymorphisms occurred in ExII, III, and VI than in ExIV and V (Fig. 4c). Examining all polymorphism classes observed in the data (Supplementary Data Table S5), the highest frequency of polymorphisms occurred in ExI and VI events and the lowest in ExV events, the majority being InDels from total polymorphism classification and numbers (Fig. 4d).

### Efficient HDR resulted in normal function of edited *MKK2*

To estimate the proper *MKK2* expression resulting from precise editing, *MKK2* functional analysis compared with WT poplars revealed regular expression (~95–100%) before salt stress in survived recovered events transferred into the greenhouse from ExII, IV, and V, and stable overexpression induced by stress application (168–173%) (Supplementary Data Fig. S17, Supplementary Data Table S6a and d). Furthermore, no detectable decrease in salt stress tolerance confirmed that *MKK2* remained functional, and no deleterious mutations occurred following HDR across exons 7, 8, and 9 (Supplementary Data Fig. S17). Concerning unidentified bands in Fig. 2d and f, events II#6, IV#90, V#37, and V#53 could not resist salinity due to *MKK2* mutations and withered. Our results showed no significant differences between surviving recovered events before and after salt stress and WT poplars in stem lengths and diameters, which validated the precise editing of the *MKK2* locus by efficient HDR within exons 7, 8, and 9 (Supplementary Data Fig. S17; Supplementary Data Table S6b, c, e, and f).

## Discussion

In contrast to gene mutation via CRISPR-Cas9 systems, precise gene targeting and knock-in mutations are more challenging but necessary for a versatile tool for research and breeding in crops and woody plants. One of the primary hindrances to this CRISPR application is the low efficiency and success of introducing genes of interest into plant genomes using traditional CRISPR constructs. Applying knowledge of pathway components regulating HDR and NHEJ mechanisms linked to introducing genes via CRISPR, here we examined the effects of the overexpression of HDR cofactors (*CtIP* and *MRE11*) [15] and disruption of NHEJ promoter *XRCC4* [18] on knock-in efficiency using *MKK2* as a case study. The importance of the *MKK2* gene in plant protection has already been shown against several environmental stress situations, including salt [23] and low temperature [29]. Since it had been previously reported [7] that HDR efficiency is directly related to the amount of DDPs present at the S/G2 cell division phases, for the first time we used a pathogenic suspension with an OD_600_ of 2.5 (~2 × 10^9^ cells ml^−1^), and the ratio of 4:1 pDDP/pgRNA to encourage the increase in DDP fragments during S/G2 cell division [15] and to avoid off-target editing caused by the extra accumulation of pgRNA [30]. Regarding this strategy, the on-target efficiency was increased up to 2-fold by targeting the *MKK2* locus and up to 4-fold by targeting the *XRCC4* locus in the designed ExV. On the other hand, the CRISPR-influenced cleavage mismatches that happened through *MKK2* editing were decreased up to 2.5-fold and were nearly zero for targeting *XRCC4* in ExV.

Several researches have shown efforts to increase HDR efficiency. One study improved HDR efficiency by 10-fold in tomatoes by integrating the 35S promoter upstream of the *ANT1* gene [2]. Another study in mammals exhibited promoted HDR efficiency with at least a 2-fold increase in HDR and a 6-fold increase in HDR/NHEJ ratio [15]. While HDR efficiency was improved to 25.7% in the *Xenopus tropicalis* genome by inserting small pieces of DNA [31], knock-in has been carried out with a 400 bp DDP into the tomato genome with low efficiency of 1.29% [32]. Moreover, some recent studies could increase HDR efficiency to 38% in mouse lines by applying multiple sgRNAs [33] and 61.5% in sheep utilizing single-strand oligodeoxynucleotides [34]. To date, several publications on *Populus* genome editing have been limited to knocking out genes and mutations caused by Cas9 and Cas12a [13, 25, 35]. In respect of enhancing HDR efficiency among improved HDR factor expressions [36], overexpression of *CtIP* and *MRE11* would improve HDR efficiency up to 19-fold [15] as well as suppressing NHEJ factors [37]. In *Arabidopsis*, knocking out the Ku 70 and Lig 4 proteins resulted in a 16- and 4-fold increase in HDR [20]. Still, no report has been carried out on improving HDR efficiency in poplar. In this study, we considered inhibiting *XRCC4* expression as one Lig 4 cofactor [21] to increase HDR efficiency and to generate a recombinant genome in poplars using an HDR system. This study combined the upregulated HDR factors CtIP and MRE11 with downregulated NHEJ cofactor XRCC4 to improve HDR efficiency by up to 48%.

The expression of *XRCC4* anywhere more than HDR-related genes *CtIP* and *MRE11* resulted in loss of HDR and led to the formation of mutant events or retention of WT. The events with expression of *XRCC4* slightly less than *CtIP* and *MRE11* expression caused the development of partial FAM signals. Conversely, events with significantly less expression of *XRCC4* than *CtIP* and *MRE11* led to promotion of fully edited FAM signals or recovered events. Finally, the only *XRCC4* deficiency could not significantly increase the expressions of HDR cofactors but somewhat improved the HDR DNA repair system.

NHEJ is characterized by introducing small irregular InDels into the targeted site. However, regardless of this mutagenic potential and its propensity for error, NHEJ plays a dominant role in repairing genome integrity, suppressing chromosomal translocations, and the bulk of repair events in the genome [38]. Also, it has been shown that silencing NHEJ factors such as Ku 70 and Ku 80 causes significantly reduced InDel rates, from 64 to 38 and 39.4%, respectively [37]. In this study, we proved that regular editing (ExI events) exhibited the highest numbers of polymorphisms, significantly more than upregulated HDR factors CtIP and MRE11 (ExIV events) and also upregulated both HDR factors CtIP and MRE11 with downregulated NHEJ factors (ExV events). We also proved that the *XRCC4* deficiency as the candidate of NHEJ combined with upregulated HDR factors promoted HDR and decreased InDels considerably via ExV events compared with the other experiments.

In addition, it has been shown that *MAPK* genes direct cellular responses against abiotic stresses such as salinity [24, 39]. Another study also reported that the *MKK2* family genes play vital roles in maize development [40]. Thus, a lack of *MKK2* expression may reduce plant stem length and diameter. This study found no significant differences in stem lengths and diameters before and after salt stress among the regular expression of *MKK2*, resulting in significantly more efficient HDR in ExV than the other experiments.

## Conclusions

In summary, we have demonstrated that NHEJ factor deficiency together with HDR factor overexpression caused meaningful enhancement of HDR efficiency, therefore greatly expanding our capacity to improve hereditary developments in poplar. We also proved that a significant reduction in CRISPR-induced polymorphisms could be achieved by following this guideline, besides improving HDR efficiency. This breakthrough technology will likely encourage biotechnological research, breeding programs, forest conservation of tree species, and development of crops.

## Materials and methods

### Targets and protein detection

The *MKK2* gene from *Populus trichocarpa* (POPTR_0018s05420g; chromosome 18) was selected as a target for editing because of its vital role in transcriptional regulation against environmental stresses. The Uniprot database (https://www.uniprot.org/) was used to download the MKK2 protein sequence, and we then used the BLAST database of the National Center for Biotechnology Information (NCBI) (https://blast.ncbi.nlm.nih.gov/) to download full DNA sequences and CDSs. To detect targets, Geneious Prime^®^ 2020.1.1 was used to analyze the *MKK2* locus and detect targets relative to the whole genome of *P. trichocarpa* downloaded from NCBI (Supplementary Data Table S1) [26, 27]. Geneious Prime was also used to analyze the *XRCC4* (POPTR_0010s08650g, chromosome 10) gene for knocking out. The PAM motif target sequences were associated with exon 8 from *MKK2* and exon 1 from *XRCC4*.

### Design of experiments and construct transformation

#### Design of experiments

This study was based on promoting HDR efficiency in poplar using designed gRNA to target the *MKK2* locus on the *P. trichocarpa* genome transferred by the pGREB31 plasmid (pgRNA) (Supplementary Data Fig. S2a), and plasmids (pgCtIP and pgMR) to target the *MKK2* locus and overexpressing *CtIP* and *MRE11*, respectively (Supplementary Data Fig. S2b and c). The other plasmid (pgCtMR) was designed to target the *MKK2* locus and overexpress both *CtIP* and *MRE11* (Supplementary Data Fig. S2d). A further plasmid (pggCtMR) was designed to carry gRNA to target NHEJ factor XRCC4, besides targeting the *MKK2* locus and overexpressing HDR factors CtIP and MRE11 (Supplementary Data Fig. S1e). The final plasmid (pgg) was designed to target both *MKK2* and *XRCC4* with no support from HDR cofactor overexpression (Supplementary Data Fig. S1f). The designed plasmids were then used in six experiments: ExI including pgRNA, ExII including pgCtIP, ExIII including pgMR, ExIV including pgCtMR, ExV including pggCtMR, and ExVI including pgg.

#### Construction of DDPs and pDDPs

To produce DDPs (Supplementary Data Fig. S1), five fragments were designed, constructed, and ligated (Supplementary Data Fig. S18a). To construct fragment 1, the OsU3 promoter and gRNA scaffold were isolated from pRGEB31 (Supplementary Data Table S7, OS1-F and -R) flanked by HindIII and BamHI sites. To increase the amount of DDP in the cell nucleus and improve HDR efficiency, the cleavage property of Cas9 was harnessed by designing two special gRNA targets, 1 and 2 (no on- and -off-targets in the whole poplar genome and only detecting special targets besides DDP) besides the DDP [41] (Supplementary Data Fig. S1). Special gRNA oligos (Sgo1-F and -R) (Supplementary Data Fig. Sa; Supplementary Data Table S7, special gRNA oligo1-F and -R) were then designed as previously described [42] to form special gRNA target 1 (Sgt1), which was then ligated into fragment 1. To construct fragment 2, we isolated 400-bp nucleotides upstream of the target from the poplar genome (5′ homology arm) (Supplementary Data Table S7, ′ Ho-F-1 and -R-1). Then, regular PCR was carried out using primers with the extensions of BamHI-special target 1 (St1) and 39 bp from complemented 5′ of fragment 3 (Supplementary Data Table S7, ′ Ho-F-2 and -R-2) (Supplementary Data Fig. S18a). To construct fragment 3, we isolated the *BleoR* CDS (Zeocin resistance gene) from the PCR^®^-XL-Topo^®^ vector (Supplementary Data Table S7, *BleoR*-1092F and 2276R). Then, overlap PCR was performed (Supplementary Data Table S7, BP1,2,3-F and -R) using the isolated *BleoR* CDS as a template to include sequences rather than remaining nucleotides from exon 8 (Leu-Ala-Thr-Leu-Lys-Thr-Cys) and exon 9 (Val-Leu-Val-Lys-Met) for adding to the 5′ *BleoR* CDS region and also 18 bp 6xHis tag and 30 bp Poly-A tail for adding to the 3′ *BleoR* CDS area (Supplementary Data Figs S1 and S18a). To assemble fragment 4, we isolated 400-bp nucleotides downstream of the target from the poplar genome (3′ homology arm) (Supplementary Data Table S7, ′ Ho-F-1 and -R-1). Then, PCR was performed to extend the 3′ homology arm with 30-bp Poly-T and NcoI-special target 2 (St2) sequences (Supplementary Data Table S7, Ho-F-2 and -R-2) (Supplementary Data Fig. S18a). Finally, standard PCR was used to isolate the OsU3 promoter and gRNA scaffold from pRGEB31 (Supplementary Data Table S7, Os2-F and Os2-R). Moreover, special gRNA oligos were designed (Sgo2-F and -R) (Supplementary Data Fig. S18a; Supplementary Data Table S7, special gRNA oligo2-F and -R) again as previously described [42] to form special gRNA target 2 (Sgt2) and ligated into fragment 5.

To construct the final pDDP construct, we ligated fragments 2 and 3 using PCR (Supplementary Data Fig. S18b). For this, we designed a 39-bp overhang on fragment 2 that was complementary to the end of fragment 3 to form preliminary DDP (Supplementary Data Fig. S18b). For this reaction, PCR was prepared with 500 ng of each component. Initially, all parts were used in the PCR reaction except primers, and then the fragments were denatured at 95°C for 5 minutes, followed by two annealing and extension cycles. Next, the PCR products were allowed to anneal at 68°C to avoid non-specific hybridization amongst the long PCR products for 30 seconds, followed by extension for 1 minute at 74°C, resulting in a double-stranded template. The primers were then added for the distal ends of fragments 2 and 3, and PCR proceeded normally. The PCR products were purified and ligated into the pEASY vector for sequencing and confirmation. The preliminary DDP product was then ligated into fragment 4 as previously described and formed secondary DDP products (Supplementary Data Fig. S18b). After sequencing and confirmation, restriction cloning was used to ligate secondary DDP products to fragments 1 and 4 (Supplementary Data Fig. S18b). Briefly, we incubated a reaction including 50 ng of each digested fragment, 10× T4 DNA ligase buffer 0.5 μl, T4 DNA ligase (NEB) 1 μl, and H_2_O to 5 μl at 25°C for 4 hours, and transferred into *Escherichia coli* DH5α competent cells for sequencing and confirmation. Subsequently, the restriction cloning technique was used to merge the DDP product and pRGEB31 vector to form the pDDP vector (Supplementary Data Fig. S18b).

#### Synthesis of pgCtIP and pgMR

To design a fused CtIP and Cas9 cassette, the CaMV35S promoter, 3xFLAG, and Cas9 CDS were isolated from pRGEB31 (Supplementary Data Fig. S19a). The CtIP CDS was then obtained using RT–PCR from the *P. trichocarpa* genome (Supplementary Data Fig. S19a; Supplementary Data Table S7, CtIP-F and -R). Next, the 3′-UTR and Poly-A fragments were isolated from the pCAG-T3-hCAS-pA plasmid (Supplementary Data Fig. S19a; Supplementary Data Table S7, Poly-A-F and -R). To complete pgCtIP, CaMV35S and 3xFLAG fragments were ligated using restriction cloning to form backbone 1 (Supplementary Data Fig. S20a). Next, the isolated Cas9 and the obtained CtIP CDS were also ligated, applying restriction cloning to form backbone 2 (Supplementary Data Fig. S20a). Backbones 1 and 2 were then ligated using HindIII restriction cloning to form backbone 3 (Supplementary Data Fig. S20a). Next, the resulting backbone 3 was ligated to the assembled 3′-UTR-Poly-A using StuI restriction cloning to create the CtIP cassette (Supplementary Data Figs S20a and S19a). SdaI and PmeI restriction enzymes were then used to restrict the cloning of the CtIP cassette and pRGEB31 and assemble the pgCtIP plasmid (Supplementary Data Figs S20a and S19a).

To construct a fusion of MRE11 and Cas9, the CaMV35 promoter, 3xFLAG, Cas9, 3′-UTR, and Poly-A were isolated, as previously described (Supplementary Data Fig. S19b). The MRE11 CDS was obtained from *P. trichocarpa* total RNA, and RT–PCR was carried out as mentioned above (Supplementary Data Fig. S19b; Supplementary Data Table S7, MRE-F and R). To complete pgMR, we ligated the isolated CaMV35S and 3xFLAG fragments concerning XhoI endonuclease to form backbone 1 (Supplementary Data Fig. S20b). Backbone 2 was then constructed using the isolated Cas9 and 3′-UTR-Poly-A fragments (Supplementary Data Fig. S20b). Backbone 1, backbone 2, and MRE11 CDS products were then merged using NotI and NdeI (NEB) restriction cloning to form the MR cassette (Supplementary Data Figs S20b and S19b). Then, restriction cloning with SdaI and PmeI was used to construct the pgMR plasmid (Supplementary Data Figs S20b and S19b).

#### Synthesis of pgCtMR, pggCtMR, and pgg

To construct the CtMR cassette, we prepared all the required fragments, as described above (Supplementary Data Fig. S19c). Afterwards, CaMV35S and 3xFLAG components were merged using XhoI restriction cloning to form backbone 1 (Supplementary Data Fig. S21a). Backbone 1 and the already obtained MRE11 CDS product (Supplementary Data Table S7, MRE-F and -R) were then ligated using NotI restriction cloning to form backbone 2 (Supplementary Data Fig. S21a). The isolated Cas9 and the obtained RT–PCR product CtIP CDS were ligated using BamHI restriction cloning to form backbone 3 (Supplementary Data Fig. S21a). Backbone 3 and isolated 3′-UTR-Poly-A fragments were then used to form backbone 4 (Supplementary Data Fig. S21a). Backbones 2 and 4 were then used to construct the CtMR cassette (Supplementary Data Figs S21a and S19c), followed by SdaI and PmeI restriction cloning to ligate the CtMR cassettes into pRGEB31, forming the pgCtMR plasmid (Supplementary Data Figs S21a and S19c). To target the *XRCC4* gene and *MKK2* simultaneously*,* we designed one cassette, including both *XRCC4* [by adding one CRISPR site (located in the 5′ region of target CDS) to mutate *XRCC4* (activity score 0.415; specificity score 100%) [26, 27]] and *MKK2* gRNAs*.* PCR was then used (Supplementary Data Table S7, XR-Cass1-F and -R) to isolate the OsU3 promoter and gRNA scaffold from the pRGEB31 vector, and *MKK*2 designed oligos (Supplementary Data Table S7, *MKK2* Oligo-F and -R) were then used to ligate the *MKK2* target duplex (Supplementary Data Fig. S19d). In addition, PCR was used (Supplementary Data Table S7; XR-Cass2-F and -R) to isolate the OsU3 promoter and gRNA scaffold again. In this process, we applied *XRCC4* designed oligos (Supplementary Data Table S7; *XRCC4*-Oligo1 and -2) to ligate the *XRCC4* target duplex (Supplementary Data Fig. S19d). The resulting fragments were then cloned using KasI restriction cloning to form the *XRCC4* cassette (backbone 1) (Supplementary Data Figs S21b and S19d). The *XRCC4* cassette was then cloned into pRGEB31 using HindIII and SdaI restriction cloning to form backbone 2 (Supplementary Data Fig. S21b). Finally, SdaI and PmeI restriction cloning was used to clone the CtMR cassette into backbone 2, creating the pggCtMR plasmid (Supplementary Data Figs S21b and S19d). Validation of construct assembly was performed using PCR, cloning into pEASY T3 vector, and DNA sequencing throughout construction. To construct a plasmid harboring both *XRCC4* and *MKK2* gRNA targets, we constructed pgg by following all processes in pggCtMR construction to prepare only backbone 2 (Supplementary Data Figs S21b and 19d) with no extra CtMR cassette (Supplementary Data Fig. S2f). Validation of construct assembly was performed as for the other constructed plasmids in this research.

### Transformation and target detection

#### Plant transformation

For transformation, poplar (*P. trichocarpa*) seedlings were cultivated in a Phytotron at 23 ± 2°C under a 16/8 light/dark photoperiod [11]. To generate transgenic lines, stems from 4-week-old clones were dipped in an optimized *A. tumefaciens* suspension (OD_600_ 2.5, 120 minutes, pH ~5, acetosyringone 200 μM) [12] for 5 minutes with gentle shaking. The transformed stems were then transferred to a semi-solid woody plant medium (WPM) containing 0.05 mg/l indole-3-butyric acid (IBA), 0.006 mg/l thidiazuron (TDZ), 200 μM acetosyringone, and 0.5% (w/v) agar. Afterwards, the stimulated stems were incubated in the dark at 23°C for 2 days. The assumed transformants were then co-cultivated in selection media enriched with 0.1 mg/l IBA, 0.006 mg/l TDZ, 100 mg/l cefotaxime, 8 mg/l hygromycin, 50 mg/l Zeocin, and 0.8% (w/v) agar. Two weeks later, buds were regenerated and then subcultured independently in media containing 0.1 mg/L IBA, 0.001 mg/l TDZ, 100 mg/l cefotaxime, 8 mg/l hygromycin, 50 mg/l Zeocin, and 0.8% (w/v) agar (grown buds on Zeocin). After 6 weeks, buds were transferred to MS medium containing 0.1 mg/l IBA, 200 mg/l cefotaxime, 70 mg/l Zeocin, and 0.8% (w/v) agar to root (recovered events). Five independent transgenic lines were used for each experiment, and each line included ~10 individuals. To generate WT lines, we followed all steps mentioned above with an empty vector (pRGEB31, including only Cas9 with no target gRNA seed) to transfer and ignoring Zeocin for selection.

#### *MKK2* locus target oligo synthesis

A pair of oligos (Supplementary Data Table ; *MKK2* Oligo-F and -R) were designed flanked by BsaI adaptors for vector construction. Synthesized oligos were then ligated into pRGEB31 vectors following BsaI digestion [42] to construct pgRNA (Supplementary Data Fig. Sa). Then, all vectors were transferred into *E. coli* (DH5α) and propagated at 37°C for 8 hours (normal conditions). Vectors were then extracted using a Plasmid Midi Kit (Qiagen, USA) and confirmed by Sanger sequencing (GenScript, Nanjing).

#### *XRCC4* locus target oligo synthesis

A pair of oligos (Supplementary Data Table ; XRCC4-Oligo1 and XRCC4-Oligo2) were designed to target a site located on the CDS of the 5′ region from *XRCC4* (chromosome 10; 9 542 280–9 542 302). According to the instructions described above, the vectors pgCTMR and pgg were constructed for *XRCC4* locus targeting and confirmed by Sanger sequencing.

### Transformation, detection, and confirmation

#### Western blotting

We used western blotting to validate the successful integration of exogenous *BleoR* in the edited events genome. For extraction of proteins, 150 mg of fresh leaves of 5-week-old buds were milled in 500 μl extraction buffer (125 mM Tris, pH 6.8, 4 M urea, 5% β-mercaptoethanol, 4% w/v SDS). Centrifugation was then performed at 13 000 rpm for 10 minutes, and the supernatant was collected for gel analysis. The extracted protein was then boiled in loading buffer (24% w/v glycerol, 100 mM Tris, 0.05% w/v Bromophenol Blue, 4% v/v β-mercaptoethanol, 8% w/v SDS) for 10 minutes. The extracted protein was analyzed by SDS–PAGE and visualized using Coomassie brilliant blue R-250 staining. Western blotting was then performed as described by Sambrook *et al*. [43], using a rabbit anti-His polyclonal antibody developed in our laboratory as the primary antibody and peroxidase-conjugated goat anti-rabbit IgG (Zhongshan Biotechnique, Beijing, China) as the secondary antibody.

#### RT–PCR

We performed RT–PCR to verify the whole and precise integration of exogenous *BleoR* regarding designed primers and complete transcription of *BleoR* and *MKK2* resulting from efficient HDR. Total RNA (100 ng/ml) was extracted by TRIzol from young leaves of 5-week-old buds grown on Zeocin-containing medium and treated with DNase I to degrade all unexpected remaining DNA. Reverse transcription was then carried out using total RNA and oligo-dT primers to synthesize the first-strand cDNA using the PrimeScript One-Step RT–PCR Kit (Ver. 2, Takara Biotechnology, Dalian, China) according to the manufacturer’s instructions. Then, two RT–PCR experiments were designed to examine *MKK2* transcription and proper HDR. The first RT–PCR was intended to isolate a 920-bp fragment of the *MKK2* CDS (Supplementary Data Table , RT-F and R) with the primers designed to amplify from the 5′ region of exon 9 (15 bp) and 3′ region of exon 8 (15 bp). The purpose was to show the precise attachment of exons 8 and 9 to direct the transcription of *MKK2* correctly. A second RT–PCR was performed to isolate a 413-bp fragment of recombinant CDS (Supplementary Data Table S7, RT-F-107 and RT-R-519). The forward primer was designed from *BleoR* and the reverse primer was designed from exon 7 of *MKK2* to show the occurrence of HDR via transcription of a single mRNA from *MKK2* and *BleoR*.

#### DNA sequencing

Genomic DNA was extracted from leaves of 5-week-old buds grown on a Zeocin-containing medium using a DNeasy Plant Mini Kit (Qiagen, USA). The quality of the extracted genomic DNA (250–350 ng/μl) was determined with a BioDrop spectrophotometer (UK).

DNA sequencing was performed to evaluate and confirm the western blotting, RT–PCR, and Southern blotting results. In addition, DNA sequencing was applied to assess the kind of mutations that occurred during genome editing. For DNA sequencing, we carried out PCR using designed primers (Supplementary Data Table S7, *MKK2*-S-7F and *MKK2*-S-1139R), EasyTaq polymerase (TransGen Biotech), and 50 ng of extracted genomic DNA as a template. Desired amplicons were then cloned into pEASY T3 vector (TransGen Biotech, Beijing, China) and used for Sanger sequencing (GenScript, Nanjing, China), followed by alignment and data analysis.

#### Southern blotting

Southern blotting was designed and performed to confirm western blotting results. First, genomic DNA (500 ng) was cleaved with BamHI and HindIII at 37°C for 4 hours. The digested DNA was then used as a PCR template to label a 160-bp probe from the integrated *BleoR* CDS into the genomic DNA (Supplementary Data Table S7; S-F and -R). Digoxigenin (DIG) reagent was used for this procedure according to the manufacturer’s instructions (catalog number 11745832910; Roche, Basel, Switzerland). The PCR product was then electrophoresed on 0.8% agarose gel. Finally, the separated fragments were shifted on a Hybond N+ nylon membrane (Amersham Biosciences, Eindhoven, Netherlands).

#### Genome targeting efficiency

The T7E1 assay detects DNA heteroduplexes resulting from mismatches (CRISPR-Cas9 influenced mismatches) formed by errors, while DSBs are repaired via NHEJ and HDR pathways [44]. Extracted genomic DNA was used from all recovered events and WT poplars. Designed primers (Supplementary Data Table S7, T7EI *MKK2*-881 F and *MKK2*-1822 R) and the Phusion High-Fidelity DNA polymerase (NEB) were applied to amplify DNA fragments (out of designed homology arms) with 942 bp from WT and 1381 bp from edited events. Approximately 100 ng of purified PCR products was denatured–annealed at 95°C for 5 minutes, cooled to 25°C at 0.1 C°/second, and incubated at 25°C for 30 minutes. Five units of T7EI were used to digest the products at 37°C for 2 hours and loaded onto a 2% agarose gel (Supplementary Data Fig. S4a). ImageJ ver2 measured the density of the bands to calculate genome targeting efficiency [on-target efficiency (%) = 100 × (uncleaved density/total density) and mismatch cleavage (%) = 100× (cleaved density/total density)] (Supplementary Data Fig. S4b). The same protocol was applied using designed primers (Supplementary Data Table S7, *XRCC4*-4314 F and *XRCC4*-4628 R) to amplify DNA fragments with 315 bp genomic DNA extracted from WT and ExV events to assess *XRCC4* targeting efficiency (Supplementary Data Fig. S4c and d). The amplified on-targets of *XRCC4* were then purified and cloned into pEASY-T3 vectors using a cloning kit according to the instructions (catalog number CT301-01, Trans, China) for sequencing and genotyping (Supplementary Data Fig. S5a). *XRCC4*-detected target gRNA was blasted through the whole genome of *P. trichocarpa*, leading to detection of only one off-target with three mismatches located on locus NC_037298.1 (chromosome LGXIV) in intragenic regions. Primers (Supplementary Data Table S7, Off-F and -R) were applied for isolation and sequencing (Supplementary Data Fig. S5b; Supplementary Data Table S2). The same protocol was then applied to calculate the *XRCC4* targeting efficiency in ExVI events and WT poplars with the same primers (Supplementary Data Fig. S6a and b). On-target genotyping was then analyzed as described above (Supplementary Data Fig. S6c). Off-target detection in ExVI revealed the same mismatches and location as ExV events (Supplementary Data Fig. S6d).

#### HDR efficiency by TaqMan real-time PCR

TaqMan real-time PCR was performed to evaluate HDR efficiency. For this purpose, the TaqMan assay applying dye labels such as FAM and VIC was performed using an Applied Biosystems real-time PCR (Applied Biosystems, Thermo Scientific, USA). High-quality grown bud genomic DNA (see Southern blotting) was used as the template for running TaqMan real-time PCR. In this assay, two fluorescent markers, FAM and VIC, attached to the 5′ region of the probe, while a non-fluorescent quencher (NFQ) bound to the 3′ region (Supplementary Data Fig. S22). Therefore, we designed primers to probe two 150-bp fragments, FAM1 (Supplementary Data Table S7, FAM1-F and -R) and FAM2 (Supplementary Data Table S7, FAM2-F and -R). In detail, FAM1 could probe 114-bp nucleotides from the 5′ homology arm and 36-bp nucleotides from *BleoR*. Also, FAM2 could probe 105-bp nucleotides from the 3′ homology arm and 45-bp nucleotides from *BleoR* (Supplementary Data Fig. S22). In addition, primers (Supplementary Data Table S7, VIC-F and -R) were also designed to probe one 106-bp fragment VIC on the *actin* gene as housekeeping (Supplementary Data Fig. S22). Each event was assessed independently for FAM1 and FAM2, recording signals in quadruplicate. Replicates with recorded signals of both FAM1 and FAM2 were analyzed as fully edited. HDR efficiency (%) was then calculated by applying the following formula: [100 × (fully edited replicates/total replicates in each experiment)]. All results were then analyzed by one way ANOVA (Supplementary Data Table S3).

#### Quantitative PCR

To verify the resulting HDR efficiency and also evaluation of *BleoR* and *MKK2* expressions, we used the synthesized cDNA (see section RT–PCR) and designed primers (Supplementary Data Table S7, qPCR section) to perform real-time PCR. The FastStart Universal SYBR Green Master mix (Rox; no. 04913914001; Roche, USA) was used with three technical repeats for each event.

#### Salt stress phenotypic evaluation

Given the roles of *MKK2* in plant protection against environmental stresses [24, 39], and to confirm the exact HDR in the recovered transgenic lines, we evaluated the *MKK2* expression and phenotypic properties via salt stress tolerance relative to WT poplar. Recovered events were planted in soil and transferred to the greenhouse. After 2 weeks of acclimation in a greenhouse, total RNA was isolated from WT leaves as a control, and all transferred recovered events were studied to evaluate the *MKK2* expression by qPCR (Supplementary Data Table S7, 2817 F-*MKK2* and 2968 R-*MKK2*). All recovered events were irrigated daily with 25 mM NaCl for 1 week following acclimation to the greenhouse for salt stress response evaluation. Total RNA was extracted from leaves of surviving events to perform qPCR. Each event was evaluated in triplicate. Stem lengths (mm) and diameters (mm) were also measured before and after salt stress.

### Statistical analysis

All data were analyzed using one-way ANOVA with Turkey *post hoc* comparisons calculated by OriginPro 2018 software (Northampton, USA). Differences were considered significant when the confidence intervals presented no overlap of the mean values with an error value of 0.05.

## Acknowledgements

This project was funded by the National Key Program on Transgenic Research (2018ZX08020002) and the National Natural Science Foundation of China (31971682, 31570650, and 2045210646).

## Author contributions

A.M. conceived, planned, and coordinated the project, performed data analysis, wrote the draft, and finalized the manuscript. H.W. carried out the experiments and contributed to data analysis and curation. X.Z., J.C.F., and Z.H.C. validated and contributed to data analysis and curation, and revised and finalized the manuscript. Z.M., W.S., J.Z., and D.L. reviewed and edited the manuscript. B.G. validated and contributed to data curation, review, and editing. R.K.V., L.Y., and Q.Z. planned, coordinated, contributed to data curation, and revised and finalized the manuscript.

## Data availability

All data supporting the findings of this study are available in the article and its supplementary data. Raw Sanger sequencing data are available on Mendeley Data at 10.17632/mnrvyvt8bs.4.

## Conflict of interest

The authors declare that they have no conflict of interest.

## Supplementary data

Supplementary data is available at *Horticulture Research* online.

## Supplementary Material

Web_Material_uhac154
