# Supplementary material for: Precise exogenous insertion and sequence replacements in poplar by simultaneous HDR overexpression and NHEJ suppression using CRISPR-Cas9
Source: Hortic Res. 2022 Jul 22;9:uhac154. doi: 10.1093/hr/uhac154 (PMC9478684; doi:10.1093/hr/uhac154)
Supplement: Web_Material_uhac154 [file web_material_uhac154.zip › Supplementary Figure 1.pptx]

## Slide 1
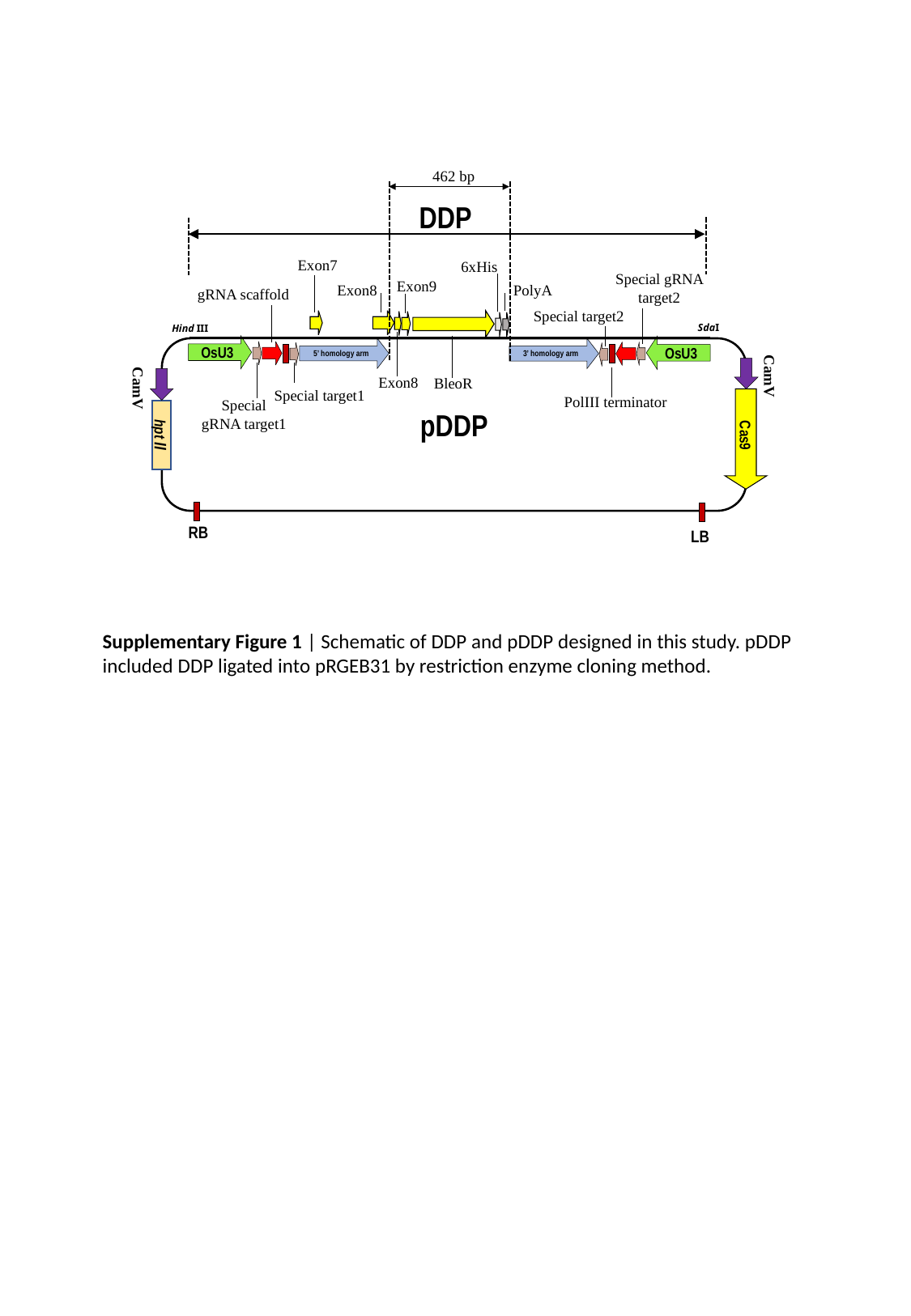

462 bp
DDP
Exon7
6xHis
Exon9
PolyA
Exon8
gRNA scaffold
OsU3
OsU3
5’ homology arm
3’ homology arm
Exon8
BleoR
Special gRNA target1
PolIII terminator
pDDP
CamV
CamV
hpt II
Cas9
RB
LB
SdaI
Hind III
Special gRNA target2
Special target2
Special target1
Supplementary Figure 1 | Schematic of DDP and pDDP designed in this study. pDDP included DDP ligated into pRGEB31 by restriction enzyme cloning method.
