# Supplementary material for: Precise exogenous insertion and sequence replacements in poplar by simultaneous HDR overexpression and NHEJ suppression using CRISPR-Cas9
Source: Hortic Res. 2022 Jul 22;9:uhac154. doi: 10.1093/hr/uhac154 (PMC9478684; doi:10.1093/hr/uhac154)
Supplement: Web_Material_uhac154 [file web_material_uhac154.zip › Supplementary Figure 14.pptx]

## Slide 1
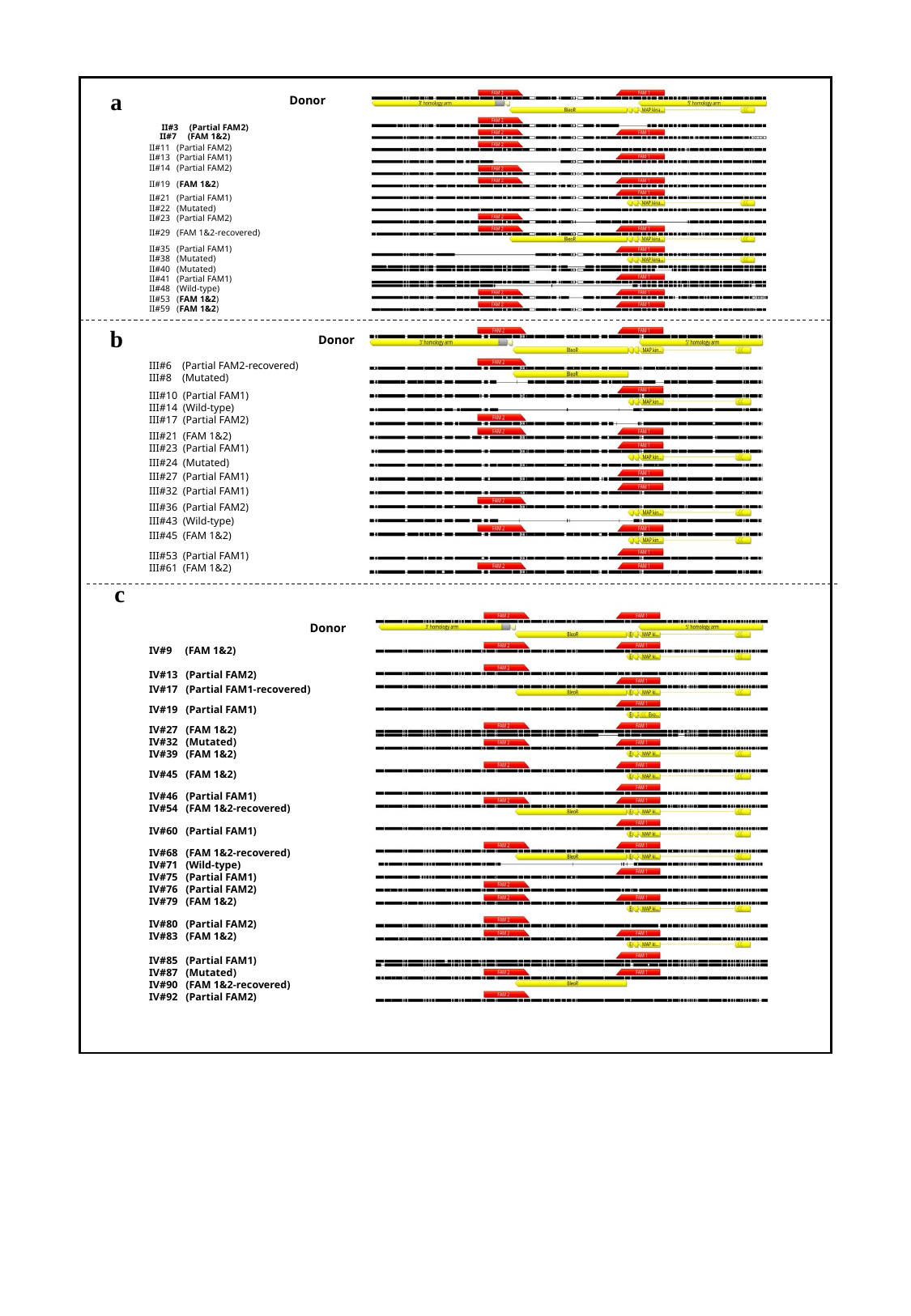

a
Donor
II#3 (Partial FAM2)
II#7 (FAM 1&2)
II#11 (Partial FAM2)
II#13 (Partial FAM1)
II#14 (Partial FAM2)
II#19 (FAM 1&2)
II#21 (Partial FAM1)
II#22 (Mutated)
II#23 (Partial FAM2)
II#29 (FAM 1&2-recovered)
II#35 (Partial FAM1)
II#38 (Mutated)
II#40 (Mutated)
II#41 (Partial FAM1)
II#48 (Wild-type)
II#53 (FAM 1&2)
II#59 (FAM 1&2)
Donor
III#6 (Partial FAM2-recovered)
III#8 (Mutated)
III#10 (Partial FAM1)
III#14 (Wild-type)
III#17 (Partial FAM2)
III#21 (FAM 1&2)
III#23 (Partial FAM1)
III#24 (Mutated)
III#27 (Partial FAM1)
III#32 (Partial FAM1)
III#36 (Partial FAM2)
III#43 (Wild-type)
III#45 (FAM 1&2)
III#53 (Partial FAM1)
III#61 (FAM 1&2)
b
c
Donor
IV#9 (FAM 1&2)
IV#13 (Partial FAM2)
IV#17 (Partial FAM1-recovered)
IV#19 (Partial FAM1)
IV#27 (FAM 1&2)
IV#32 (Mutated)
IV#39 (FAM 1&2)
IV#45 (FAM 1&2)
IV#46 (Partial FAM1)
IV#54 (FAM 1&2-recovered)
IV#60 (Partial FAM1)
IV#68 (FAM 1&2-recovered)
IV#71 (Wild-type)
IV#75 (Partial FAM1)
IV#76 (Partial FAM2)
IV#79 (FAM 1&2)
IV#80 (Partial FAM2)
IV#83 (FAM 1&2)
IV#85 (Partial FAM1)
IV#87 (Mutated)
IV#90 (FAM 1&2-recovered)
IV#92 (Partial FAM2)

## Slide 2
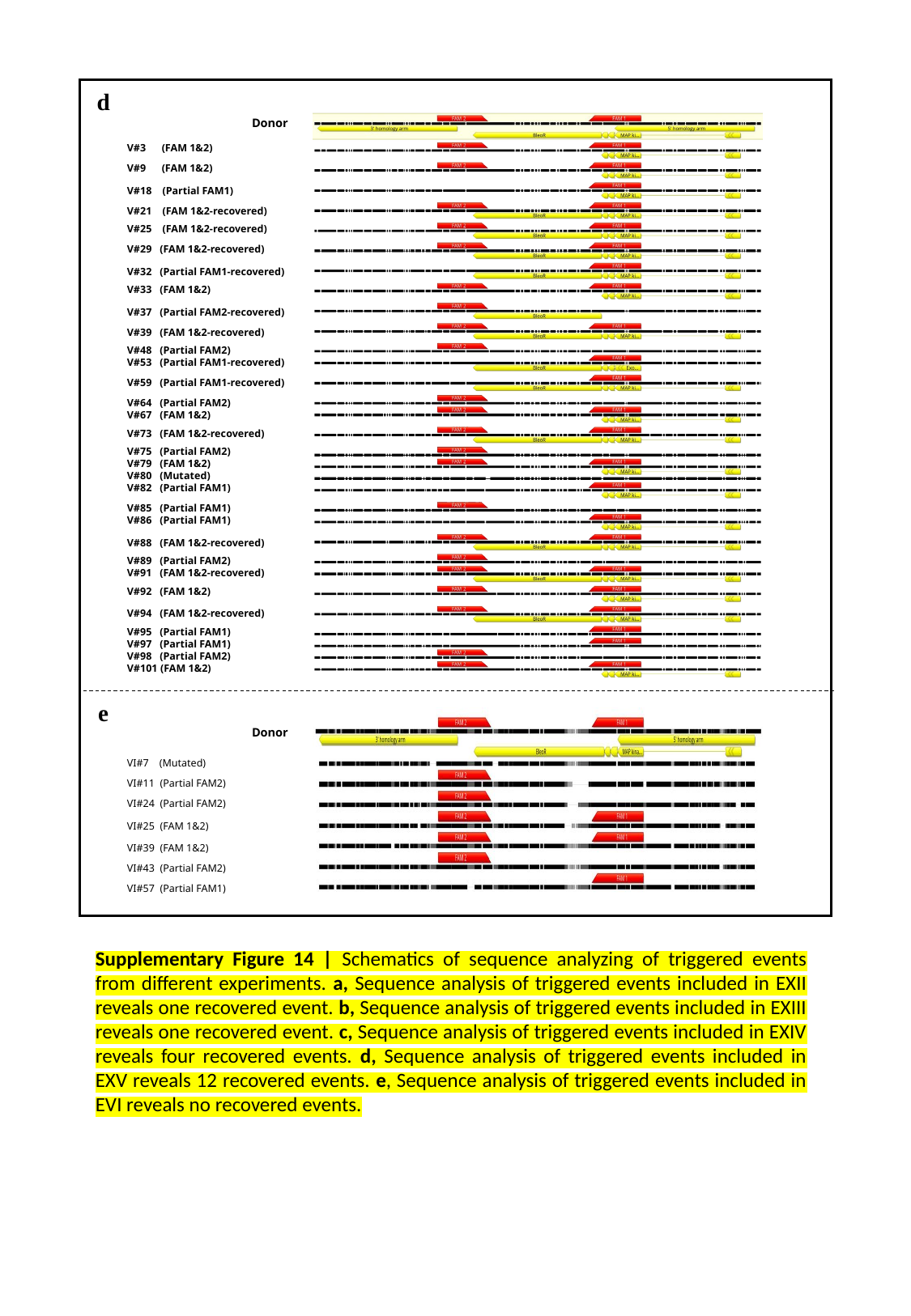

d
Donor
V#3 (FAM 1&2)
V#9 (FAM 1&2)
V#18 (Partial FAM1)
V#21 (FAM 1&2-recovered)
V#25 (FAM 1&2-recovered)
V#29 (FAM 1&2-recovered)
V#32 (Partial FAM1-recovered)
V#33 (FAM 1&2)
V#37 (Partial FAM2-recovered)
V#39 (FAM 1&2-recovered)
V#48 (Partial FAM2)
V#53 (Partial FAM1-recovered)
V#59 (Partial FAM1-recovered)
V#64 (Partial FAM2)
V#67 (FAM 1&2)
V#73 (FAM 1&2-recovered)
V#75 (Partial FAM2)
V#79 (FAM 1&2)
V#80 (Mutated)
V#82 (Partial FAM1)
V#85 (Partial FAM1)
V#86 (Partial FAM1)
V#88 (FAM 1&2-recovered)
V#89 (Partial FAM2)
V#91 (FAM 1&2-recovered)
V#92 (FAM 1&2)
V#94 (FAM 1&2-recovered)
V#95 (Partial FAM1)
V#97 (Partial FAM1)
V#98 (Partial FAM2)
V#101 (FAM 1&2)
e
Donor
VI#7 (Mutated)
VI#11 (Partial FAM2)
VI#24 (Partial FAM2)
VI#25 (FAM 1&2)
VI#39 (FAM 1&2)
VI#43 (Partial FAM2)
VI#57 (Partial FAM1)
Supplementary Figure 14 | Schematics of sequence analyzing of triggered events from different experiments. a, Sequence analysis of triggered events included in EXII reveals one recovered event. b, Sequence analysis of triggered events included in EXIII reveals one recovered event. c, Sequence analysis of triggered events included in EXIV reveals four recovered events. d, Sequence analysis of triggered events included in EXV reveals 12 recovered events. e, Sequence analysis of triggered events included in EVI reveals no recovered events.
