# Supplementary material for: Precise exogenous insertion and sequence replacements in poplar by simultaneous HDR overexpression and NHEJ suppression using CRISPR-Cas9
Source: Hortic Res. 2022 Jul 22;9:uhac154. doi: 10.1093/hr/uhac154 (PMC9478684; doi:10.1093/hr/uhac154)
Supplement: Web_Material_uhac154 [file web_material_uhac154.zip › Supplementary Figure 15.pptx]

## Slide 1
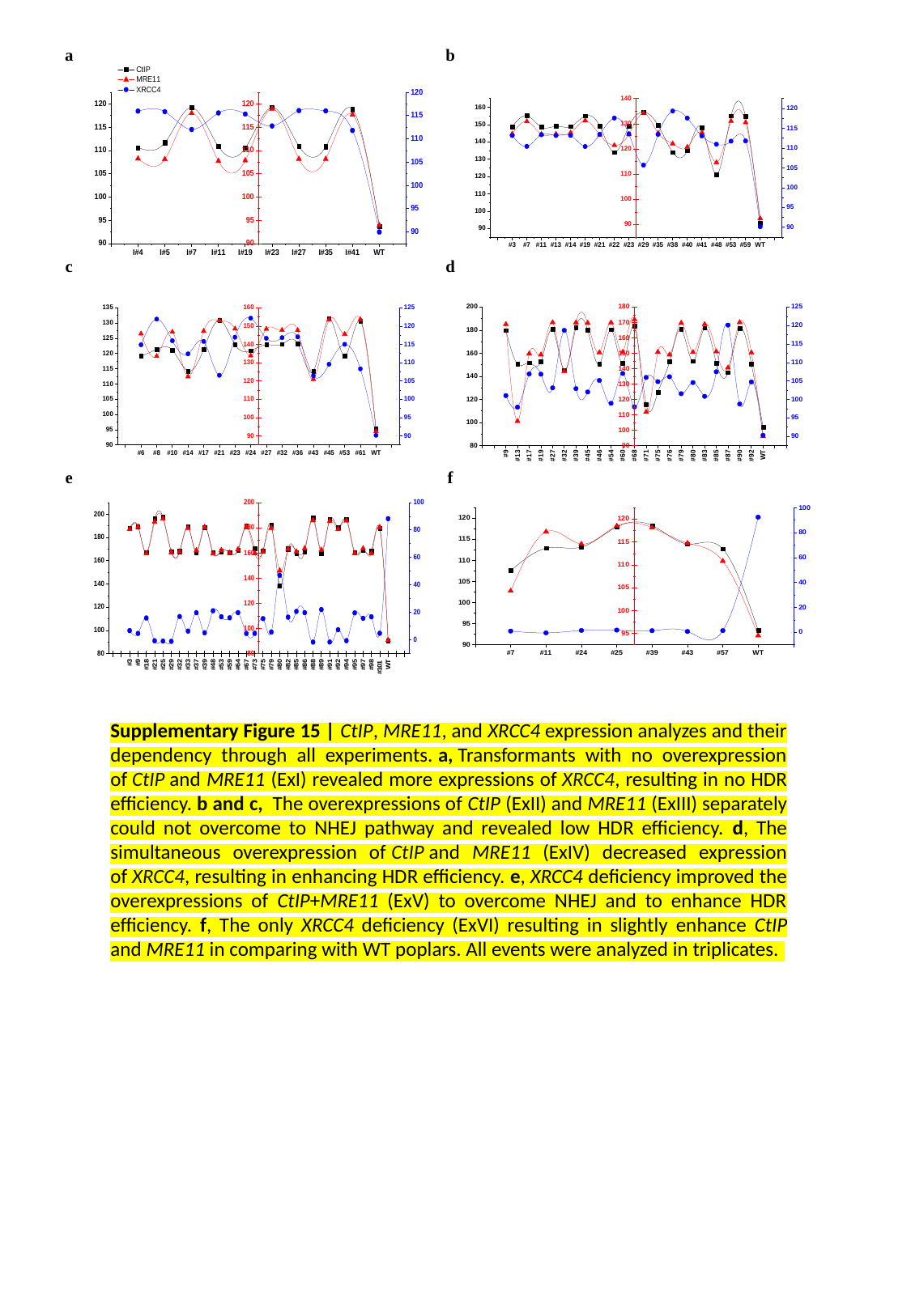

a
b
c
d
e
f
Supplementary Figure 15 | CtIP, MRE11, and XRCC4 expression analyzes and their dependency through all experiments. a, Transformants with no overexpression of CtIP and MRE11 (ExI) revealed more expressions of XRCC4, resulting in no HDR efficiency. b and c,  The overexpressions of CtIP (ExII) and MRE11 (ExIII) separately could not overcome to NHEJ pathway and revealed low HDR efficiency. d, The simultaneous overexpression of CtIP and MRE11 (ExIV) decreased expression of XRCC4, resulting in enhancing HDR efficiency. e, XRCC4 deficiency improved the overexpressions of CtIP+MRE11 (ExV) to overcome NHEJ and to enhance HDR efficiency. f, The only XRCC4 deficiency (ExVI) resulting in slightly enhance CtIP and MRE11 in comparing with WT poplars. All events were analyzed in triplicates.
