# Supplementary material for: Precise exogenous insertion and sequence replacements in poplar by simultaneous HDR overexpression and NHEJ suppression using CRISPR-Cas9
Source: Hortic Res. 2022 Jul 22;9:uhac154. doi: 10.1093/hr/uhac154 (PMC9478684; doi:10.1093/hr/uhac154)
Supplement: Web_Material_uhac154 [file web_material_uhac154.zip › Supplementary Figure 16.pptx]

## Slide 1
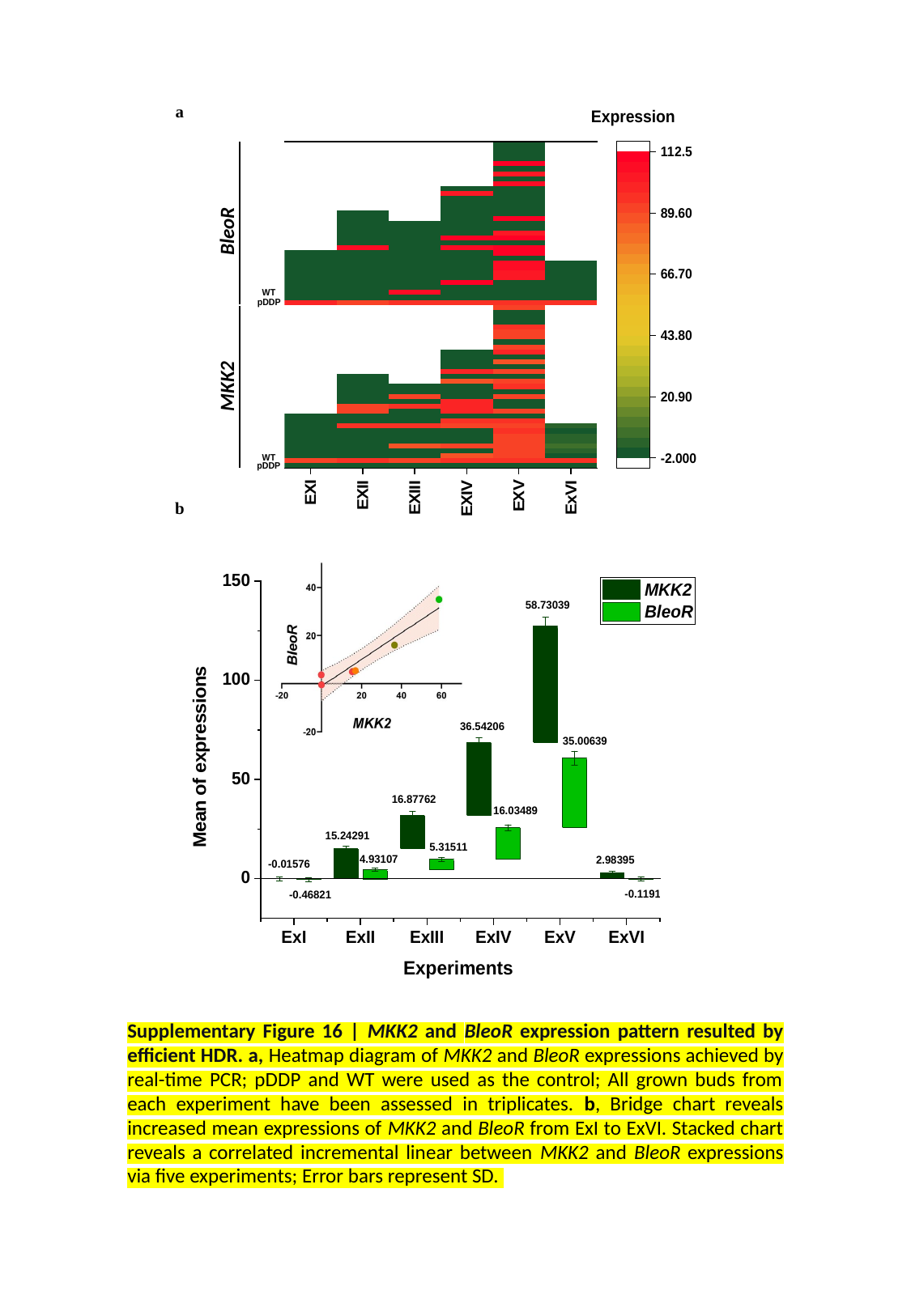

BleoR
MKK2
a
b
Supplementary Figure 16 | MKK2 and BleoR expression pattern resulted by efficient HDR. a, Heatmap diagram of MKK2 and BleoR expressions achieved by real-time PCR; pDDP and WT were used as the control; All grown buds from each experiment have been assessed in triplicates. b, Bridge chart reveals increased mean expressions of MKK2 and BleoR from ExI to ExVI. Stacked chart reveals a correlated incremental linear between MKK2 and BleoR expressions via five experiments; Error bars represent SD.
