# Supplementary material for: Precise exogenous insertion and sequence replacements in poplar by simultaneous HDR overexpression and NHEJ suppression using CRISPR-Cas9
Source: Hortic Res. 2022 Jul 22;9:uhac154. doi: 10.1093/hr/uhac154 (PMC9478684; doi:10.1093/hr/uhac154)
Supplement: Web_Material_uhac154 [file web_material_uhac154.zip › Supplementary Figure 17.pptx]

## Slide 1
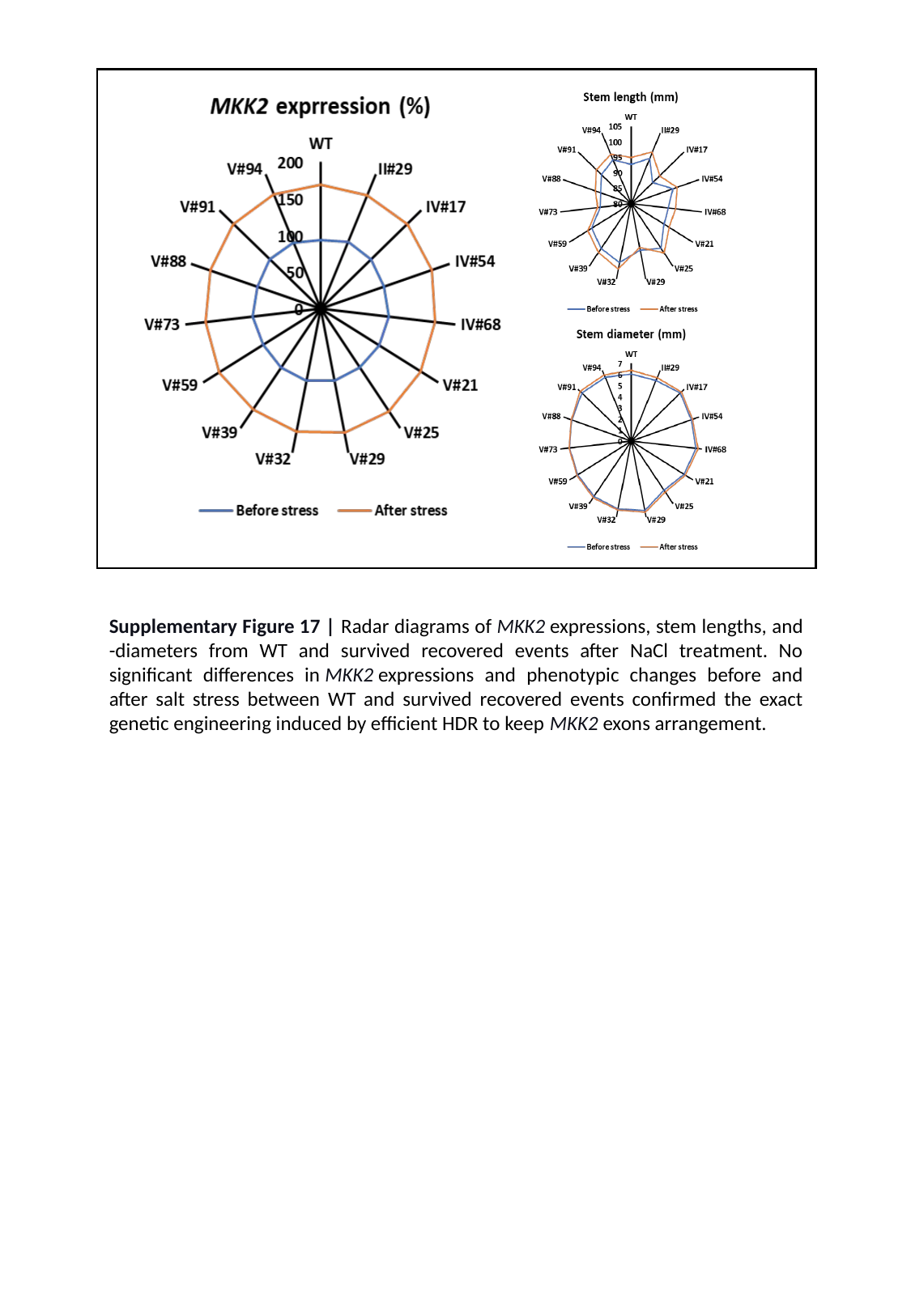

Supplementary Figure 17 | Radar diagrams of MKK2 expressions, stem lengths, and -diameters from WT and survived recovered events after NaCl treatment. No significant differences in MKK2 expressions and phenotypic changes before and after salt stress between WT and survived recovered events confirmed the exact genetic engineering induced by efficient HDR to keep MKK2 exons arrangement.
