# Supplementary material for: Precise exogenous insertion and sequence replacements in poplar by simultaneous HDR overexpression and NHEJ suppression using CRISPR-Cas9
Source: Hortic Res. 2022 Jul 22;9:uhac154. doi: 10.1093/hr/uhac154 (PMC9478684; doi:10.1093/hr/uhac154)
Supplement: Web_Material_uhac154 [file web_material_uhac154.zip › Supplementary Figure 18.pptx]

## Slide 1
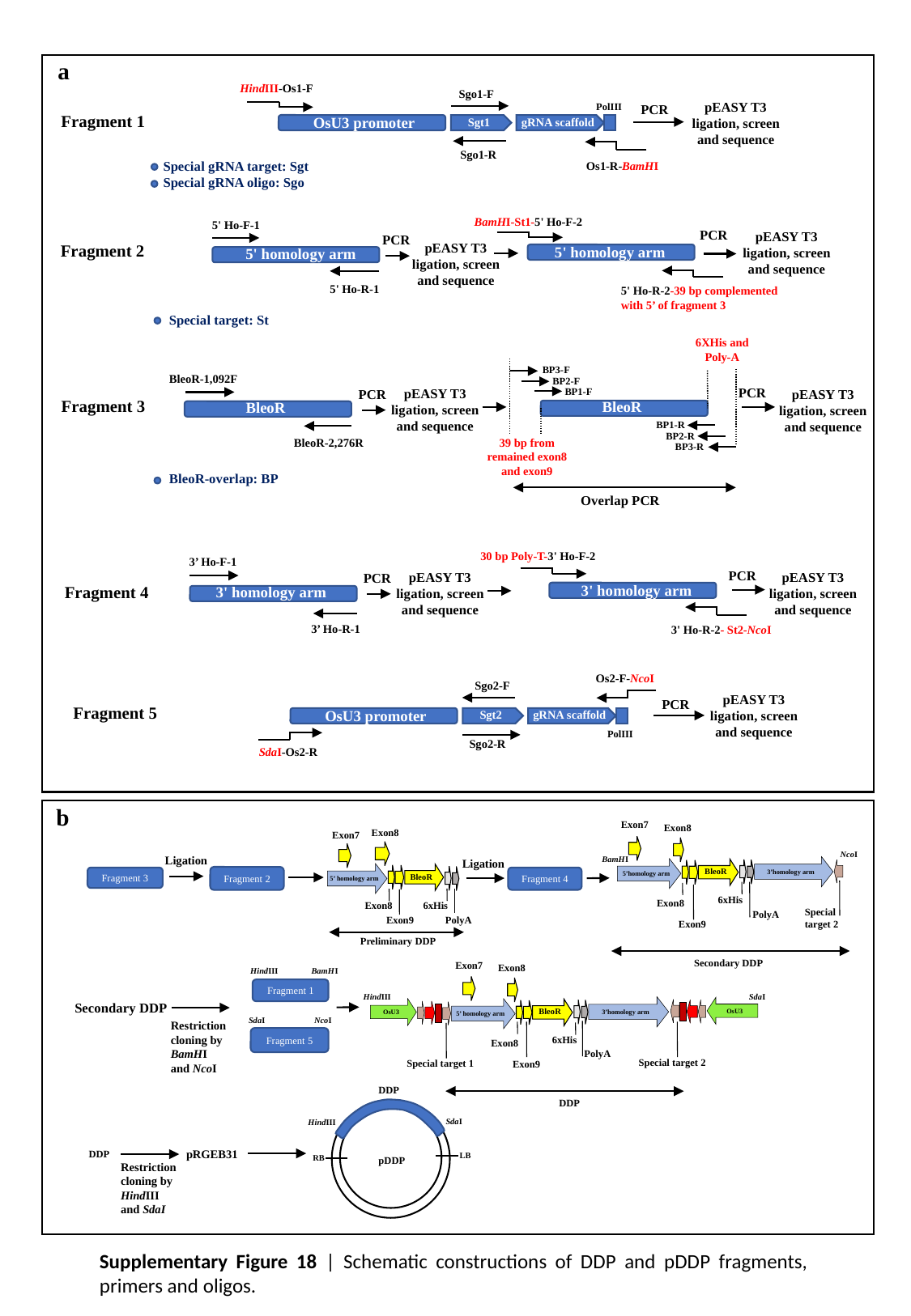

a
HindIII-Os1-F
Sgo1-F
pEASY T3 ligation, screen and sequence
PolIII
OsU3 promoter
Sgt1
gRNA scaffold
PCR
Fragment 1
Sgo1-R
Special gRNA target: Sgt
Special gRNA oligo: Sgo
Os1-R-BamHI
BamHI-St1-5' Ho-F-2
PCR
5' homology arm
5' Ho-R-2-39 bp complemented with 5’ of fragment 3
5' Ho-F-1
5' homology arm
5' Ho-R-1
Special target: St
PCR
pEASY T3 ligation, screen and sequence
pEASY T3 ligation, screen and sequence
Fragment 2
6XHis and Poly-A
BP3-F
BleoR-1,092F
BP2-F
PCR
BP1-F
pEASY T3 ligation, screen and sequence
PCR
pEASY T3 ligation, screen and sequence
BleoR
BleoR
BP1-R
BP2-R
BleoR-2,276R
39 bp from remained exon8 and exon9
BP3-R
BleoR-overlap: BP
Overlap PCR
Fragment 3
30 bp Poly-T-3' Ho-F-2
3’ Ho-F-1
PCR
pEASY T3 ligation, screen and sequence
pEASY T3 ligation, screen and sequence
PCR
3' homology arm
3' homology arm
3’ Ho-R-1
3' Ho-R-2- St2-NcoI
Fragment 4
Os2-F-NcoI
Sgo2-F
Fragment 5
OsU3 promoter
Sgt2
gRNA scaffold
PolIII
Sgo2-R
SdaI-Os2-R
pEASY T3 ligation, screen and sequence
PCR
b
Exon7
Exon8
5’homology arm
BleoR
6xHis
Exon8
PolyA
Exon9
3’homology arm
Secondary DDP
NcoI
BamHI
Special target 2
Exon8
Exon7
5’ homology arm
BleoR
6xHis
Exon8
PolyA
Exon9
Preliminary DDP
Ligation
Ligation
Fragment 2
Fragment 3
Fragment 4
Exon7
Exon8
SdaI
3’homology arm
OsU3
5’ homology arm
BleoR
6xHis
Exon8
PolyA
Special target 2
Exon9
DDP
OsU3
HindIII
Special target 1
HindIII
BamHI
Fragment 1
Secondary DDP
SdaI
NcoI
Fragment 5
Restriction cloning by BamHI and NcoI
DDP
SdaI
HindIII
LB
RB
pDDP
pRGEB31
DDP
Restriction cloning by HindIII and SdaI
Supplementary Figure 18 | Schematic constructions of DDP and pDDP fragments, primers and oligos.
