# Supplementary material for: Precise exogenous insertion and sequence replacements in poplar by simultaneous HDR overexpression and NHEJ suppression using CRISPR-Cas9
Source: Hortic Res. 2022 Jul 22;9:uhac154. doi: 10.1093/hr/uhac154 (PMC9478684; doi:10.1093/hr/uhac154)
Supplement: Web_Material_uhac154 [file web_material_uhac154.zip › Supplementary Figure 19.pptx]

## Slide 1
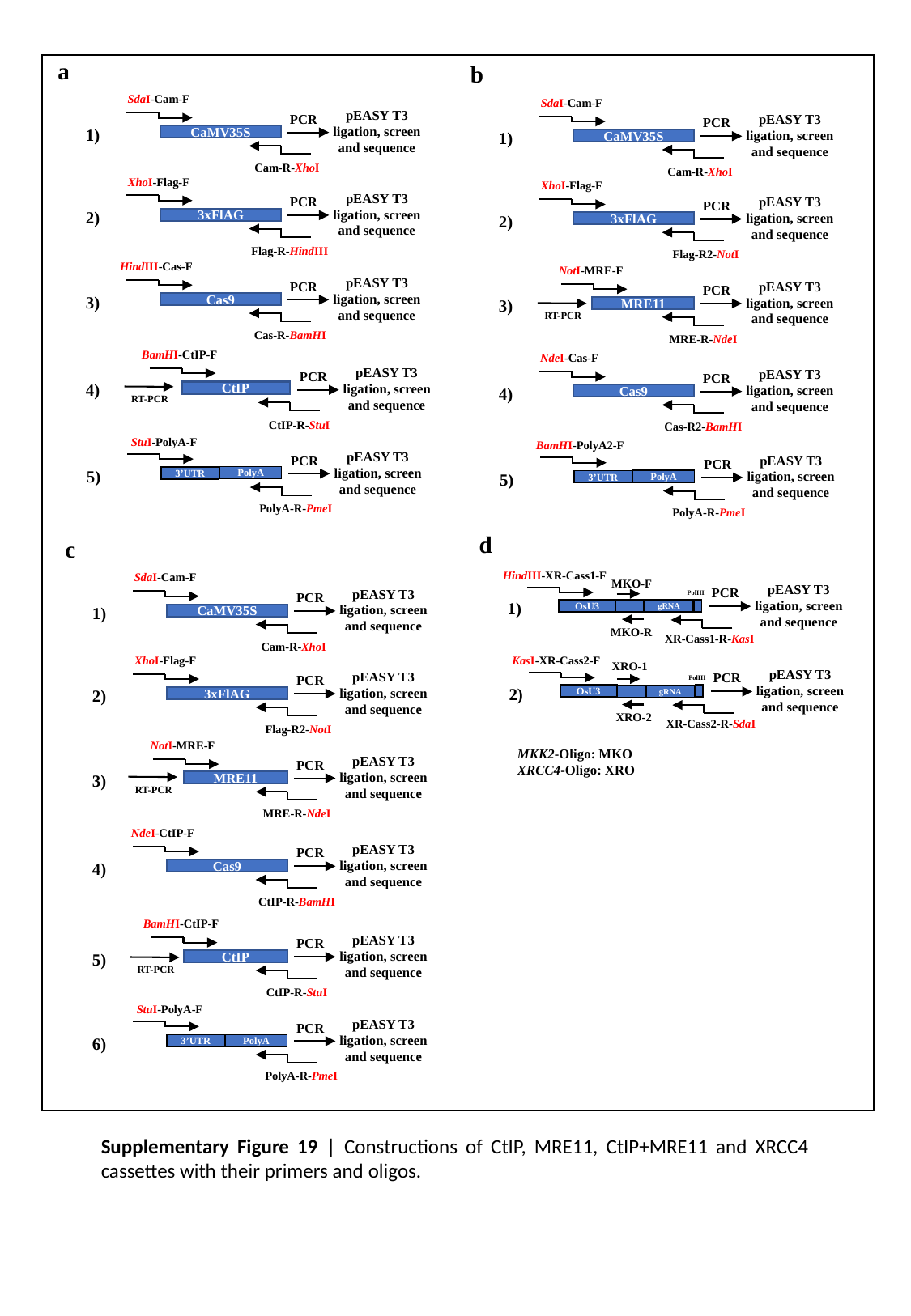

a
SdaI-Cam-F
pEASY T3 ligation, screen and sequence
PCR
1)
CaMV35S
Cam-R-XhoI
XhoI-Flag-F
pEASY T3 ligation, screen and sequence
PCR
2)
3xFlAG
Flag-R-HindIII
HindIII-Cas-F
pEASY T3 ligation, screen and sequence
PCR
3)
Cas9
Cas-R-BamHI
BamHI-CtIP-F
CtIP
RT-PCR
CtIP-R-StuI
pEASY T3 ligation, screen and sequence
PCR
4)
StuI-PolyA-F
pEASY T3 ligation, screen and sequence
PCR
5)
3’UTR
PolyA-R-PmeI
PolyA
b
SdaI-Cam-F
pEASY T3 ligation, screen and sequence
PCR
1)
CaMV35S
Cam-R-XhoI
XhoI-Flag-F
pEASY T3 ligation, screen and sequence
PCR
2)
3xFlAG
Flag-R2-NotI
NotI-MRE-F
MRE11
RT-PCR
MRE-R-NdeI
pEASY T3 ligation, screen and sequence
PCR
3)
NdeI-Cas-F
pEASY T3 ligation, screen and sequence
PCR
4)
Cas9
Cas-R2-BamHI
BamHI-PolyA2-F
pEASY T3 ligation, screen and sequence
PCR
5)
3’UTR
PolyA-R-PmeI
PolyA
d
HindIII-XR-Cass1-F
OsU3
gRNA
XR-Cass1-R-KasI
PolIII
pEASY T3 ligation, screen and sequence
PCR
1)
KasI-XR-Cass2-F
OsU3
gRNA
XR-Cass2-R-SdaI
PolIII
pEASY T3 ligation, screen and sequence
PCR
2)
MKO-F
MKO-R
XRO-1
XRO-2
MKK2-Oligo: MKO
XRCC4-Oligo: XRO
c
SdaI-Cam-F
pEASY T3 ligation, screen and sequence
PCR
1)
CaMV35S
Cam-R-XhoI
XhoI-Flag-F
pEASY T3 ligation, screen and sequence
PCR
2)
3xFlAG
Flag-R2-NotI
NotI-MRE-F
MRE11
RT-PCR
MRE-R-NdeI
pEASY T3 ligation, screen and sequence
PCR
3)
NdeI-CtIP-F
pEASY T3 ligation, screen and sequence
PCR
4)
Cas9
CtIP-R-BamHI
BamHI-CtIP-F
CtIP
CtIP-R-StuI
RT-PCR
pEASY T3 ligation, screen and sequence
PCR
5)
StuI-PolyA-F
pEASY T3 ligation, screen and sequence
PCR
6)
3’UTR
PolyA-R-PmeI
PolyA
Supplementary Figure 19 | Constructions of CtIP, MRE11, CtIP+MRE11 and XRCC4 cassettes with their primers and oligos.
