# Supplementary material for: Precise exogenous insertion and sequence replacements in poplar by simultaneous HDR overexpression and NHEJ suppression using CRISPR-Cas9
Source: Hortic Res. 2022 Jul 22;9:uhac154. doi: 10.1093/hr/uhac154 (PMC9478684; doi:10.1093/hr/uhac154)
Supplement: Web_Material_uhac154 [file web_material_uhac154.zip › Supplementary Figure 2.pptx]

## Slide 1
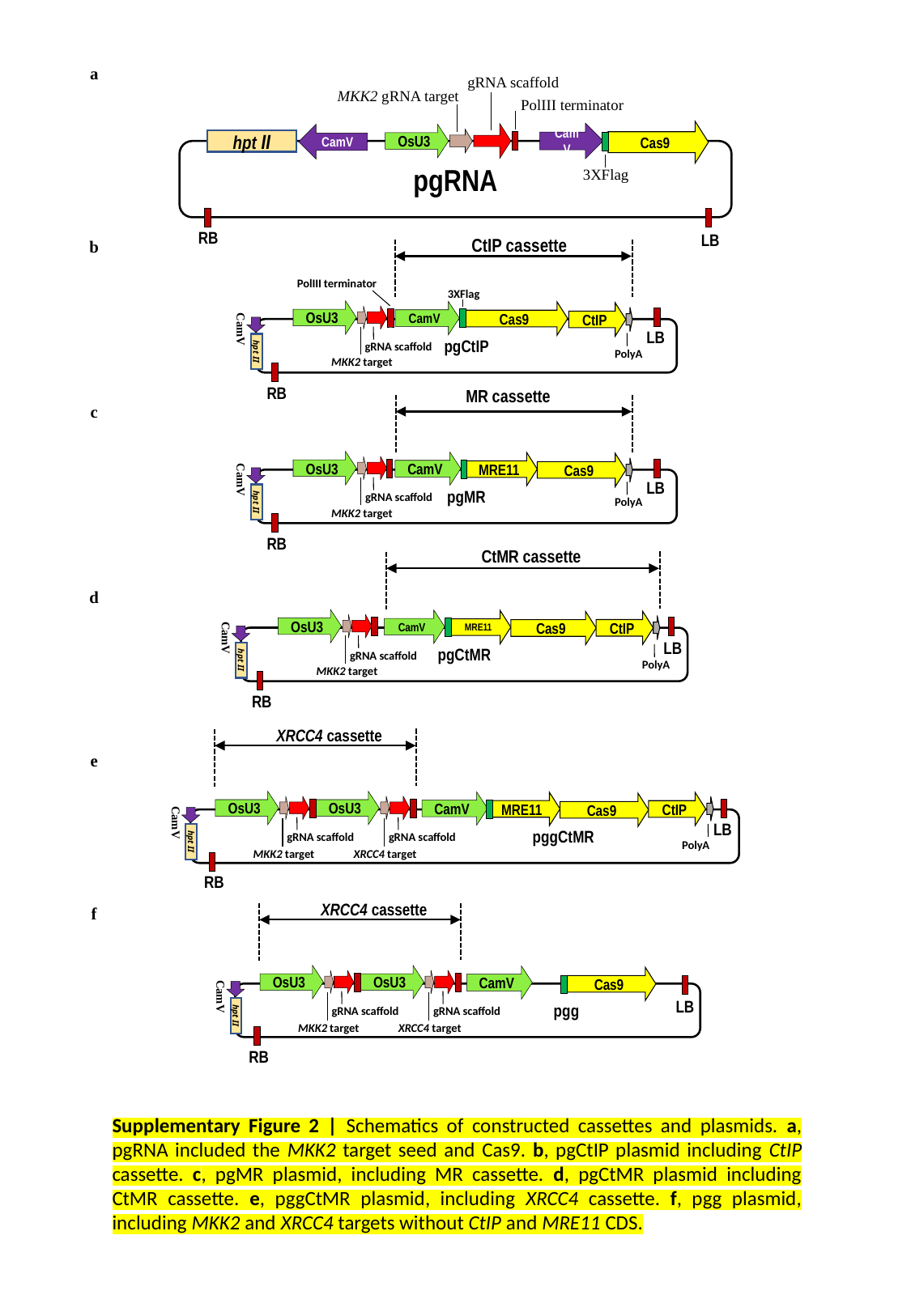

a
gRNA scaffold
MKK2 gRNA target
PolIII terminator
hpt II
CamV
CamV
Cas9
OsU3
pgRNA
RB
LB
3XFlag
CtIP cassette
PolIII terminator
3XFlag
OsU3
CamV
Cas9
CtIP
pgCtIP
CamV
hpt II
LB
RB
MKK2 target
gRNA scaffold
PolyA
b
MR cassette
OsU3
CamV
MRE11
Cas9
pgMR
CamV
hpt II
LB
RB
MKK2 target
gRNA scaffold
PolyA
c
CtMR cassette
OsU3
CamV
MRE11
Cas9
pgCtMR
CamV
hpt II
LB
RB
MKK2 target
gRNA scaffold
PolyA
CtIP
d
XRCC4 cassette
OsU3
OsU3
CamV
MRE11
CtIP
Cas9
 pggCtMR
CamV
gRNA scaffold
MKK2 target
gRNA scaffold
XRCC4 target
LB
hpt II
PolyA
RB
e
XRCC4 cassette
OsU3
OsU3
CamV
Cas9
 pgg
CamV
gRNA scaffold
MKK2 target
gRNA scaffold
XRCC4 target
LB
hpt II
RB
f
Supplementary Figure 2 | Schematics of constructed cassettes and plasmids. a, pgRNA included the MKK2 target seed and Cas9. b, pgCtIP plasmid including CtIP cassette. c, pgMR plasmid, including MR cassette. d, pgCtMR plasmid including CtMR cassette. e, pggCtMR plasmid, including XRCC4 cassette. f, pgg plasmid, including MKK2 and XRCC4 targets without CtIP and MRE11 CDS.
