# Supplementary material for: Precise exogenous insertion and sequence replacements in poplar by simultaneous HDR overexpression and NHEJ suppression using CRISPR-Cas9
Source: Hortic Res. 2022 Jul 22;9:uhac154. doi: 10.1093/hr/uhac154 (PMC9478684; doi:10.1093/hr/uhac154)
Supplement: Web_Material_uhac154 [file web_material_uhac154.zip › Supplementary Figure 20.pptx]

## Slide 1
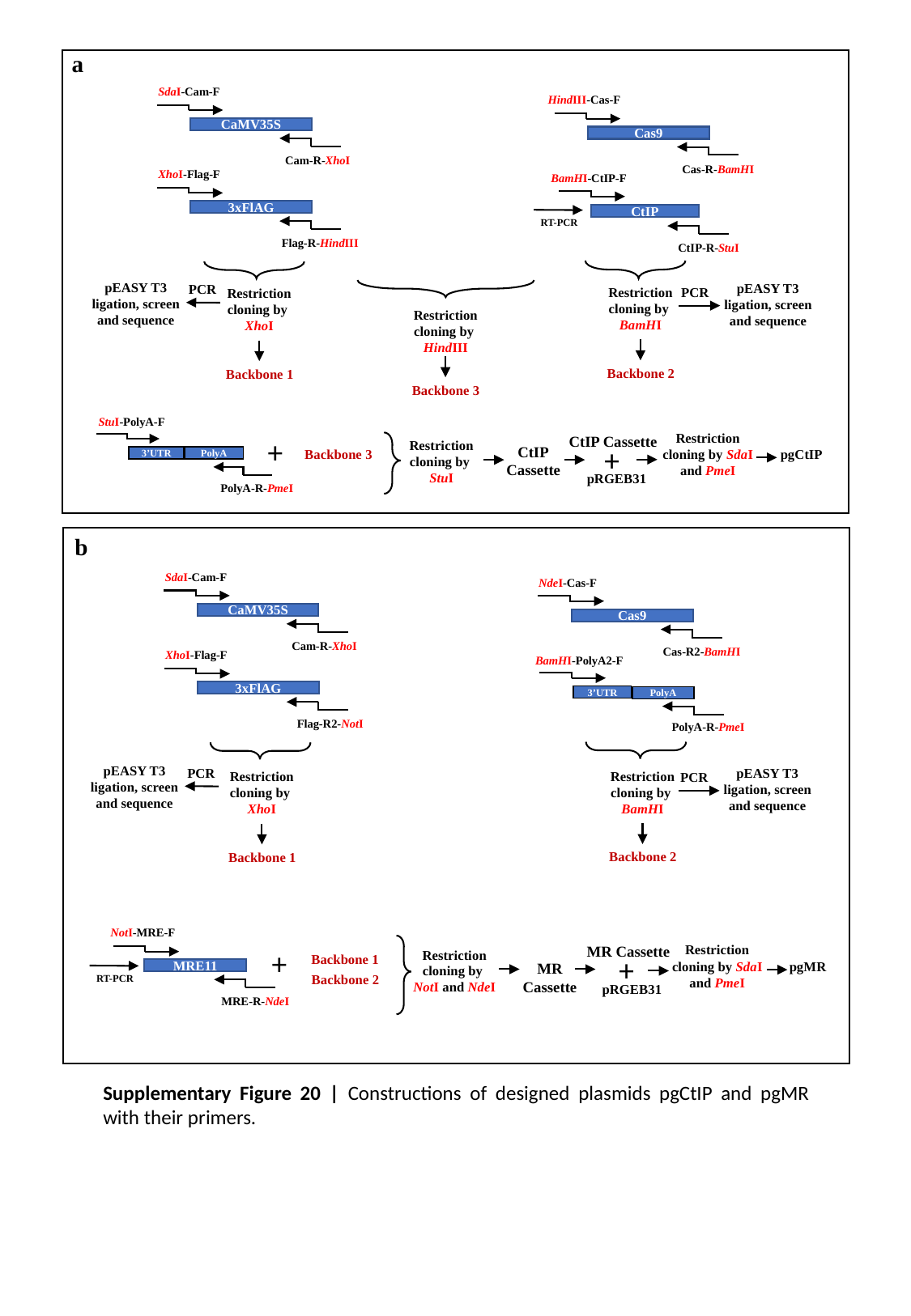

a
SdaI-Cam-F
CaMV35S
Cam-R-XhoI
HindIII-Cas-F
Cas9
Cas-R-BamHI
XhoI-Flag-F
3xFlAG
Flag-R-HindIII
BamHI-CtIP-F
CtIP
RT-PCR
CtIP-R-StuI
Restriction cloning by
BamHI
Backbone 2
Restriction cloning by
XhoI
Backbone 1
pEASY T3 ligation, screen and sequence
PCR
pEASY T3 ligation, screen and sequence
PCR
Restriction cloning by
HindIII
Backbone 3
StuI-PolyA-F
3’UTR
PolyA
PolyA-R-PmeI
Restriction cloning by SdaI and PmeI
CtIP Cassette
pRGEB31
pgCtIP
+
Restriction cloning by
StuI
CtIP
Cassette
Backbone 3
+
SdaI-Cam-F
CaMV35S
Cam-R-XhoI
XhoI-Flag-F
3xFlAG
Flag-R2-NotI
NdeI-Cas-F
Cas9
Cas-R2-BamHI
BamHI-PolyA2-F
3’UTR
PolyA-R-PmeI
PolyA
pEASY T3 ligation, screen and sequence
PCR
pEASY T3 ligation, screen and sequence
Restriction cloning by
BamHI
Backbone 2
Restriction cloning by
XhoI
Backbone 1
PCR
NotI-MRE-F
MRE11
RT-PCR
MRE-R-NdeI
Restriction cloning by
NotI and NdeI
Restriction cloning by SdaI and PmeI
MR Cassette
pgMR
pRGEB31
+
Backbone 1
MR
Cassette
Backbone 2
b
+
Supplementary Figure 20 | Constructions of designed plasmids pgCtIP and pgMR with their primers.
