# Supplementary material for: Precise exogenous insertion and sequence replacements in poplar by simultaneous HDR overexpression and NHEJ suppression using CRISPR-Cas9
Source: Hortic Res. 2022 Jul 22;9:uhac154. doi: 10.1093/hr/uhac154 (PMC9478684; doi:10.1093/hr/uhac154)
Supplement: Web_Material_uhac154 [file web_material_uhac154.zip › Supplementary Figure 21.pptx]

## Slide 1
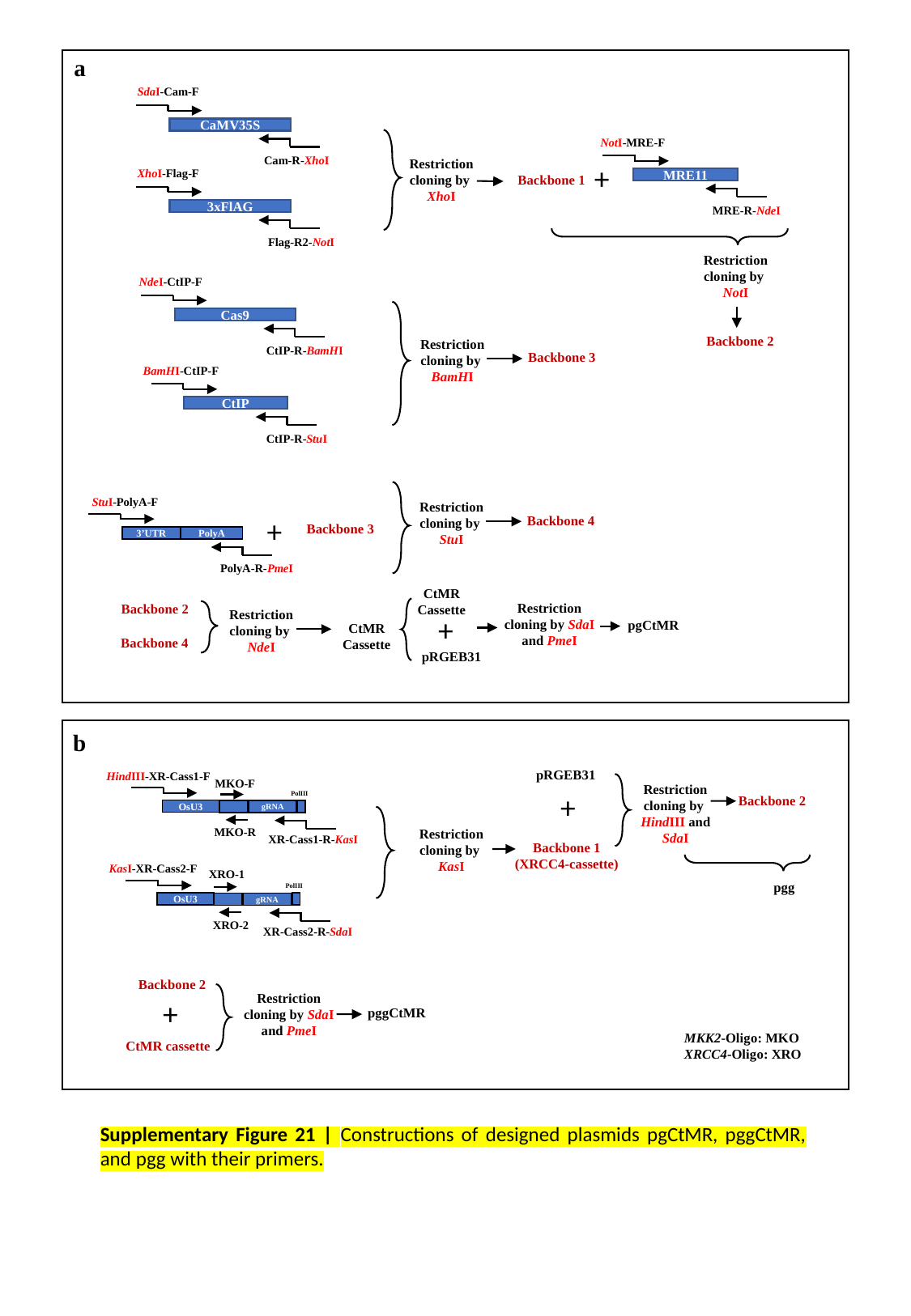

a
SdaI-Cam-F
CaMV35S
Cam-R-XhoI
NotI-MRE-F
MRE11
MRE-R-NdeI
Restriction cloning by
XhoI
+
XhoI-Flag-F
3xFlAG
Flag-R2-NotI
Backbone 1
Restriction cloning by
NotI
Backbone 2
NdeI-CtIP-F
Cas9
CtIP-R-BamHI
Restriction cloning by
BamHI
Backbone 3
BamHI-CtIP-F
CtIP
CtIP-R-StuI
StuI-PolyA-F
3’UTR
PolyA-R-PmeI
PolyA
Restriction cloning by
StuI
Backbone 4
+
Backbone 3
CtMR
Cassette
Restriction cloning by SdaI and PmeI
Backbone 2
Restriction cloning by
NdeI
+
pgCtMR
CtMR
Cassette
Backbone 4
pRGEB31
b
pRGEB31
HindIII-XR-Cass1-F
OsU3
gRNA
XR-Cass1-R-KasI
PolIII
MKO-F
MKO-R
Restriction cloning by
HindIII and SdaI
Backbone 2
Restriction cloning by
KasI
Backbone 1
(XRCC4-cassette)
KasI-XR-Cass2-F
OsU3
gRNA
XR-Cass2-R-SdaI
PolIII
XRO-1
XRO-2
Backbone 2
Restriction cloning by SdaI and PmeI
pggCtMR
CtMR cassette
MKK2-Oligo: MKO
XRCC4-Oligo: XRO
+
pgg
+
Supplementary Figure 21 | Constructions of designed plasmids pgCtMR, pggCtMR, and pgg with their primers.
