# Supplementary material for: Precise exogenous insertion and sequence replacements in poplar by simultaneous HDR overexpression and NHEJ suppression using CRISPR-Cas9
Source: Hortic Res. 2022 Jul 22;9:uhac154. doi: 10.1093/hr/uhac154 (PMC9478684; doi:10.1093/hr/uhac154)
Supplement: Web_Material_uhac154 [file web_material_uhac154.zip › Supplementary Figure 22.pptx]

## Slide 1
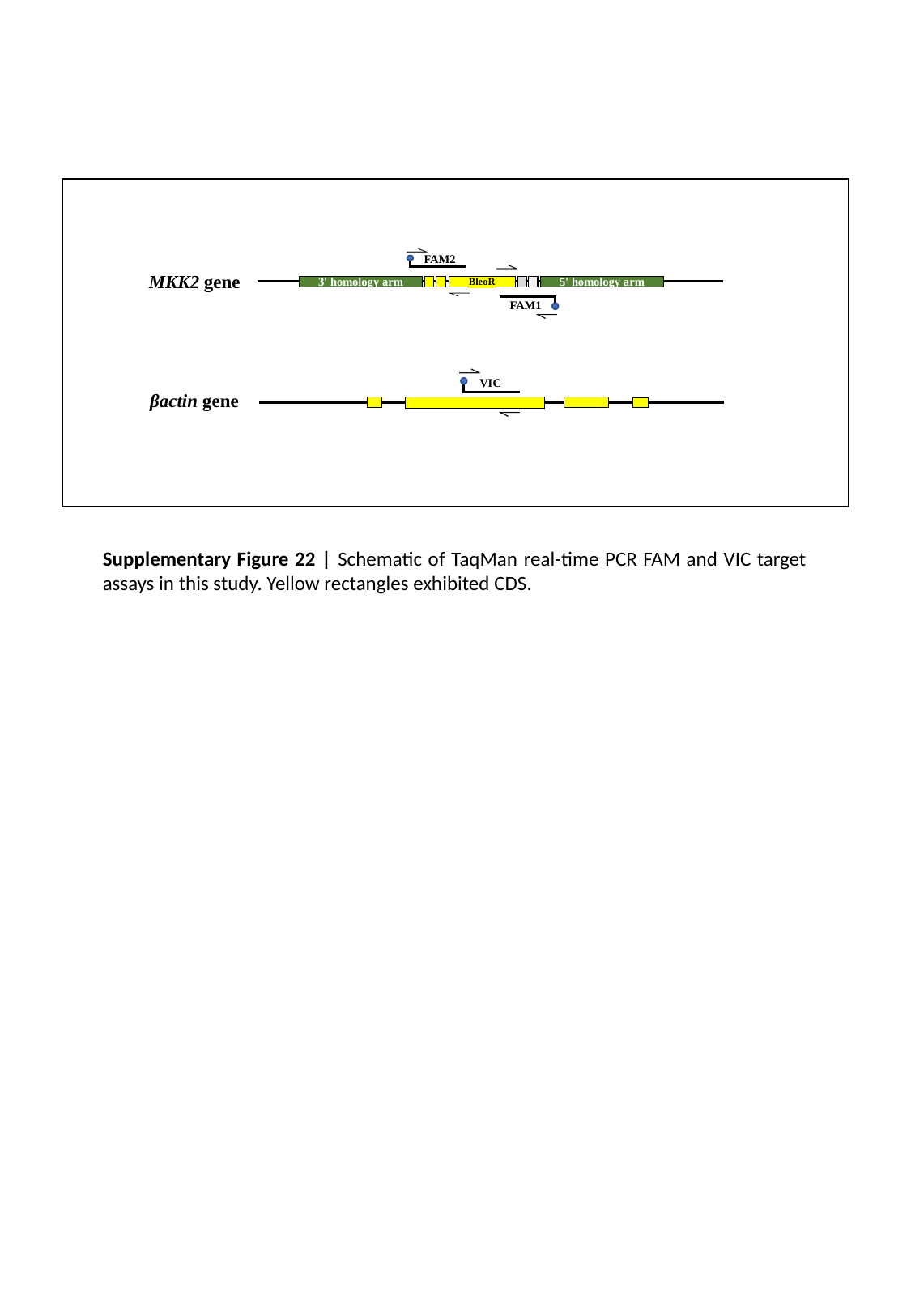

FAM2
FAM1
MKK2 gene
3' homology arm
BleoR
5' homology arm
VIC
βactin gene
Supplementary Figure 22 | Schematic of TaqMan real-time PCR FAM and VIC target assays in this study. Yellow rectangles exhibited CDS.
