# Supplementary material for: Precise exogenous insertion and sequence replacements in poplar by simultaneous HDR overexpression and NHEJ suppression using CRISPR-Cas9
Source: Hortic Res. 2022 Jul 22;9:uhac154. doi: 10.1093/hr/uhac154 (PMC9478684; doi:10.1093/hr/uhac154)
Supplement: Web_Material_uhac154 [file web_material_uhac154.zip › Supplementary Figure 3.pptx]

## Slide 1
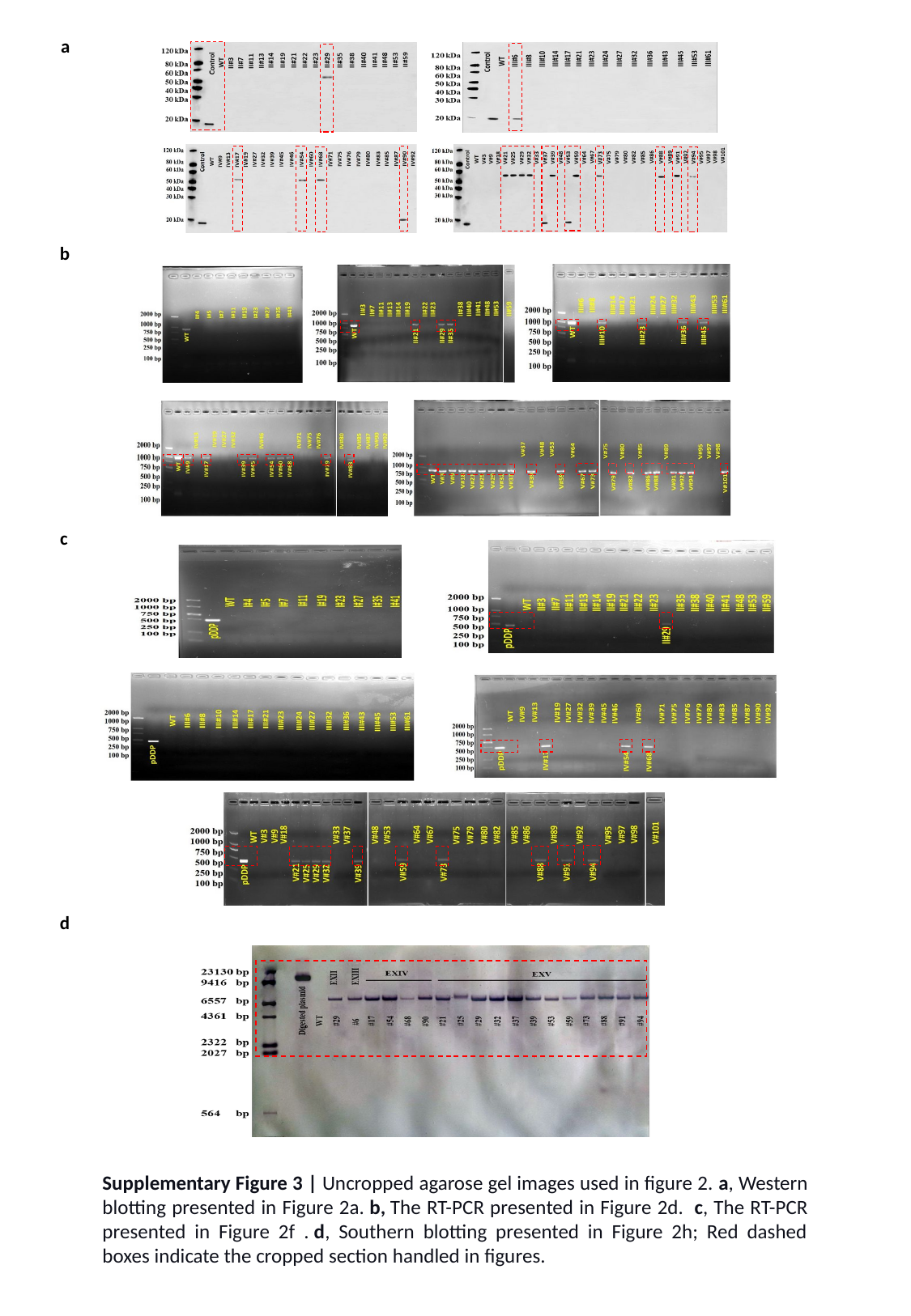

a
b
c
d
Supplementary Figure 3 | Uncropped agarose gel images used in figure 2. a, Western blotting presented in Figure 2a. b, The RT-PCR presented in Figure 2d.  c, The RT-PCR presented in Figure 2f . d, Southern blotting presented in Figure 2h; Red dashed boxes indicate the cropped section handled in figures.
