# Supplementary material for: Precise exogenous insertion and sequence replacements in poplar by simultaneous HDR overexpression and NHEJ suppression using CRISPR-Cas9
Source: Hortic Res. 2022 Jul 22;9:uhac154. doi: 10.1093/hr/uhac154 (PMC9478684; doi:10.1093/hr/uhac154)
Supplement: Web_Material_uhac154 [file web_material_uhac154.zip › Supplementary Figure 4.pptx]

## Slide 1
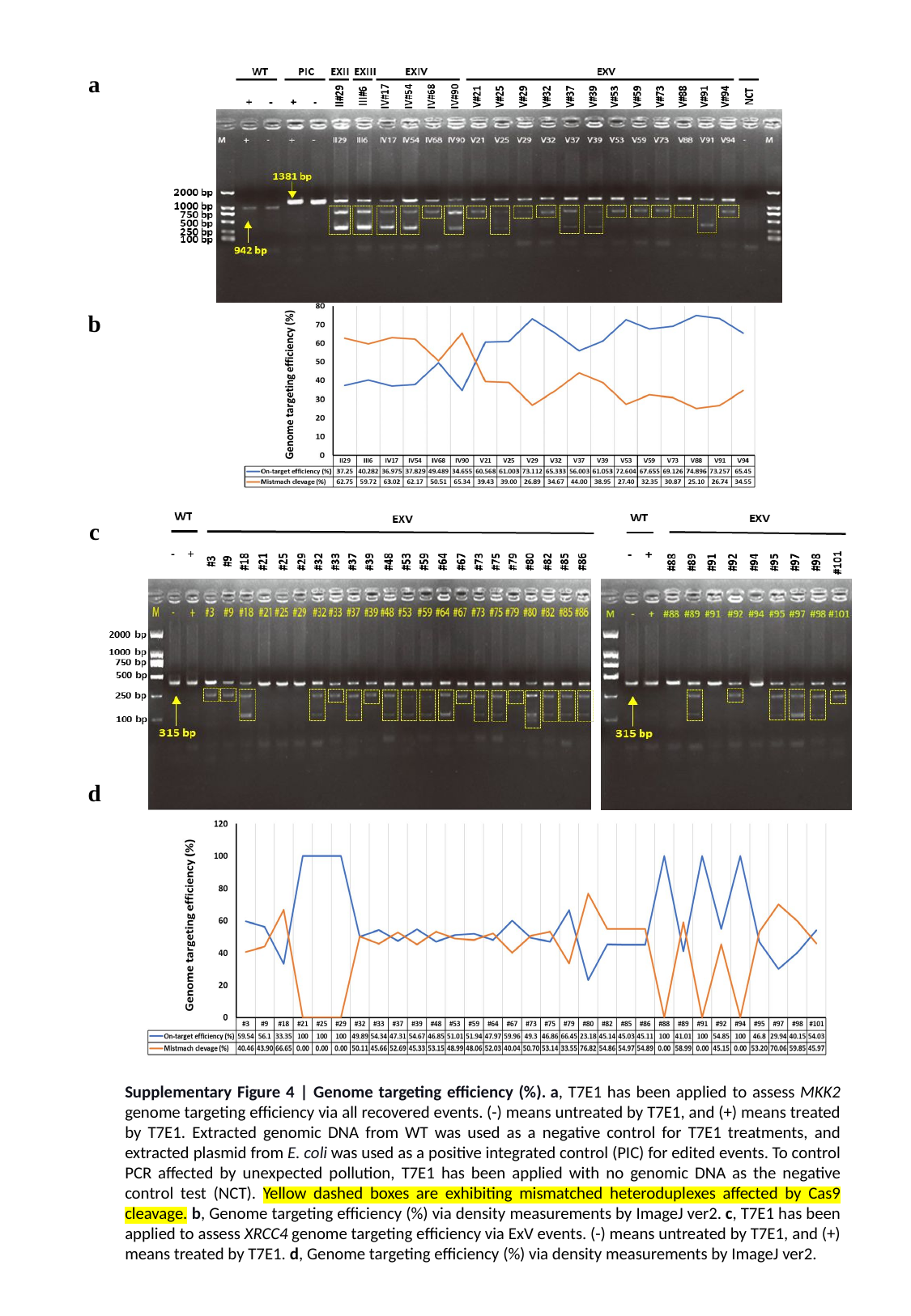

a
b
c
d
Supplementary Figure 4 | Genome targeting efficiency (%). a, T7E1 has been applied to assess MKK2 genome targeting efficiency via all recovered events. (-) means untreated by T7E1, and (+) means treated by T7E1. Extracted genomic DNA from WT was used as a negative control for T7E1 treatments, and extracted plasmid from E. coli was used as a positive integrated control (PIC) for edited events. To control PCR affected by unexpected pollution, T7E1 has been applied with no genomic DNA as the negative control test (NCT). Yellow dashed boxes are exhibiting mismatched heteroduplexes affected by Cas9 cleavage. b, Genome targeting efficiency (%) via density measurements by ImageJ ver2. c, T7E1 has been applied to assess XRCC4 genome targeting efficiency via ExV events. (-) means untreated by T7E1, and (+) means treated by T7E1. d, Genome targeting efficiency (%) via density measurements by ImageJ ver2.
