# Supplementary material for: Precise exogenous insertion and sequence replacements in poplar by simultaneous HDR overexpression and NHEJ suppression using CRISPR-Cas9
Source: Hortic Res. 2022 Jul 22;9:uhac154. doi: 10.1093/hr/uhac154 (PMC9478684; doi:10.1093/hr/uhac154)
Supplement: Web_Material_uhac154 [file web_material_uhac154.zip › Supplementary Figure 5.pptx]

## Slide 1
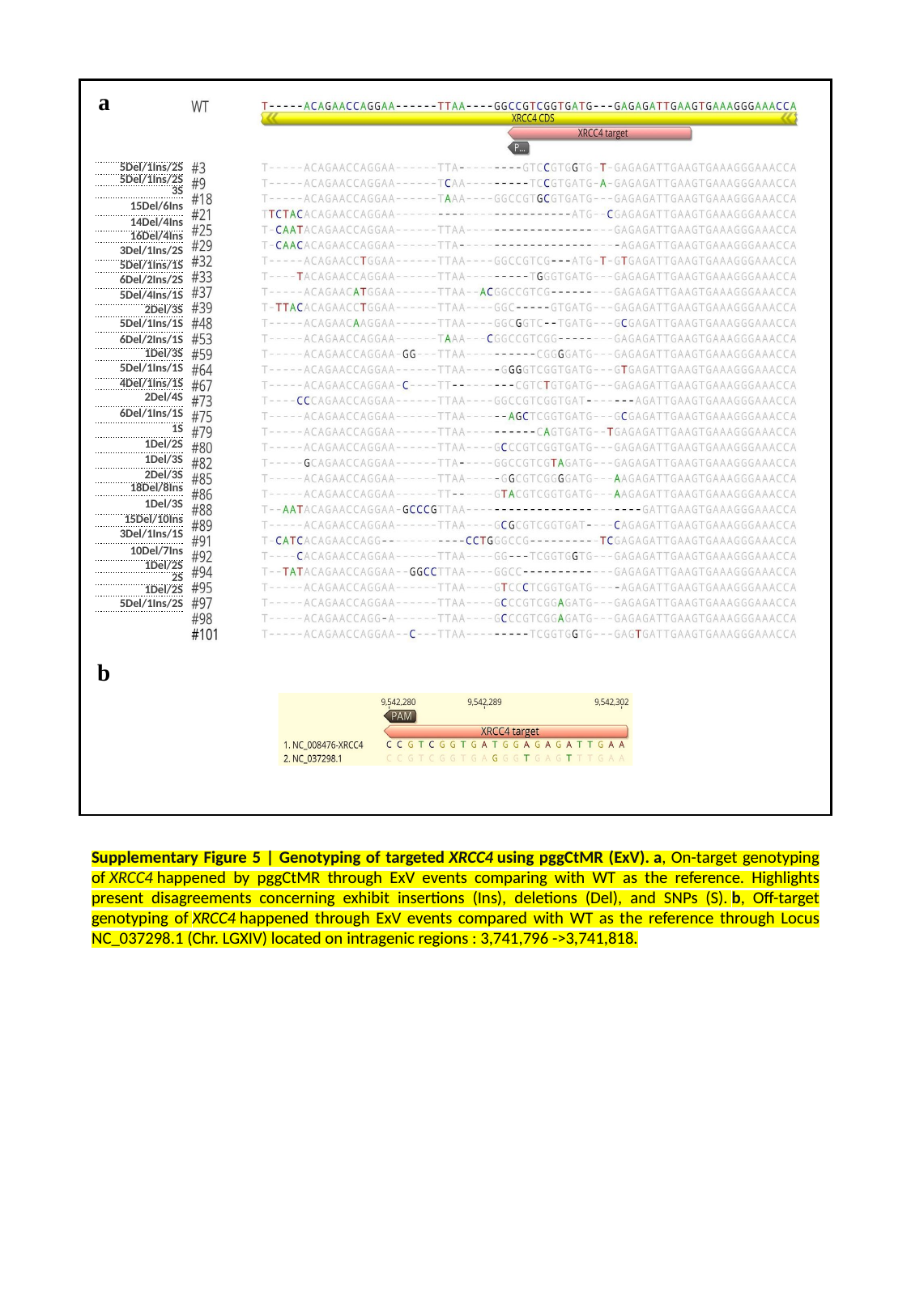

a
| 5Del/1Ins/2S |
| --- |
| 5Del/1Ins/2S |
| 3S |
| 15Del/6Ins |
| 14Del/4Ins |
| 16Del/4Ins |
| 3Del/1Ins/2S |
| 5Del/1Ins/1S |
| 6Del/2Ins/2S |
| 5Del/4Ins/1S |
| 2Del/3S |
| 5Del/1Ins/1S |
| 6Del/2Ins/1S |
| 1Del/3S |
| 5Del/1Ins/1S |
| 4Del/1Ins/1S |
| 2Del/4S |
| 6Del/1Ins/1S |
| 1S |
| 1Del/2S |
| 1Del/3S |
| 2Del/3S |
| 18Del/8Ins |
| 1Del/3S |
| 15Del/10Ins |
| 3Del/1Ins/1S |
| 10Del/7Ins |
| 1Del/2S |
| 2S |
| 1Del/2S |
| 5Del/1Ins/2S |
b
Supplementary Figure 5 | Genotyping of targeted XRCC4 using pggCtMR (ExV). a, On-target genotyping of XRCC4 happened by pggCtMR through ExV events comparing with WT as the reference. Highlights present disagreements concerning exhibit insertions (Ins), deletions (Del), and SNPs (S). b, Off-target genotyping of XRCC4 happened through ExV events compared with WT as the reference through Locus NC_037298.1 (Chr. LGXIV) located on intragenic regions : 3,741,796 ->3,741,818.
