# Supplementary material for: Precise exogenous insertion and sequence replacements in poplar by simultaneous HDR overexpression and NHEJ suppression using CRISPR-Cas9
Source: Hortic Res. 2022 Jul 22;9:uhac154. doi: 10.1093/hr/uhac154 (PMC9478684; doi:10.1093/hr/uhac154)
Supplement: Web_Material_uhac154 [file web_material_uhac154.zip › Supplementary Figure 6.pptx]

## Slide 1
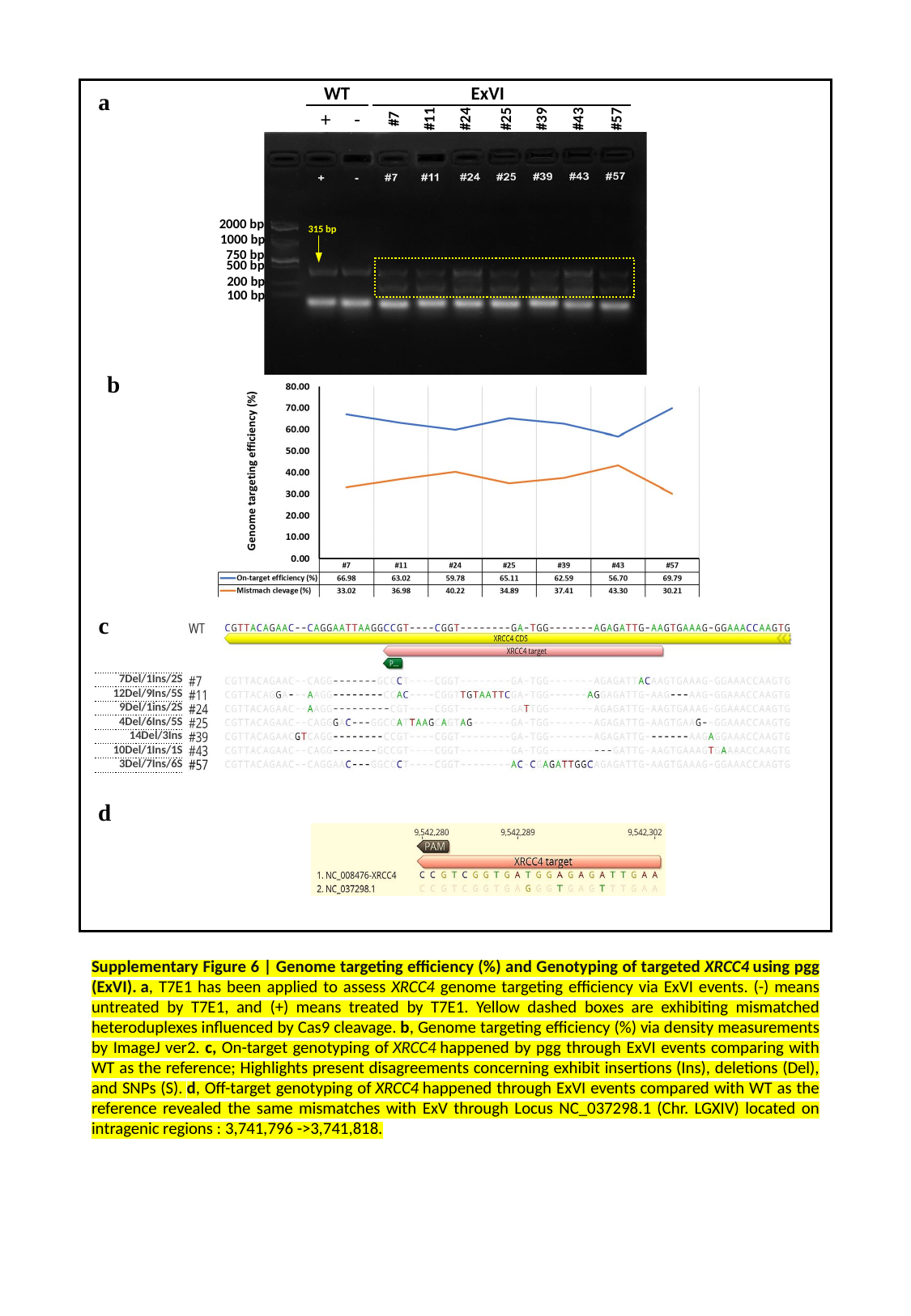

WT
ExVI
#11
#24
#25
#39
#43
#57
#7
+ -
2000 bp
1000 bp
750 bp
500 bp
200 bp
100 bp
315 bp
a
b
c
| 7Del/1Ins/2S |
| --- |
| 12Del/9Ins/5S |
| 9Del/1ins/2S |
| 4Del/6Ins/5S |
| 14Del/3Ins |
| 10Del/1Ins/1S |
| 3Del/7Ins/6S |
| |
| |
| |
d
Supplementary Figure 6 | Genome targeting efficiency (%) and Genotyping of targeted XRCC4 using pgg (ExVI). a, T7E1 has been applied to assess XRCC4 genome targeting efficiency via ExVI events. (-) means untreated by T7E1, and (+) means treated by T7E1. Yellow dashed boxes are exhibiting mismatched heteroduplexes influenced by Cas9 cleavage. b, Genome targeting efficiency (%) via density measurements by ImageJ ver2. c, On-target genotyping of XRCC4 happened by pgg through ExVI events comparing with WT as the reference; Highlights present disagreements concerning exhibit insertions (Ins), deletions (Del), and SNPs (S). d, Off-target genotyping of XRCC4 happened through ExVI events compared with WT as the reference revealed the same mismatches with ExV through Locus NC_037298.1 (Chr. LGXIV) located on intragenic regions : 3,741,796 ->3,741,818.
