# Supplementary material for: Precise exogenous insertion and sequence replacements in poplar by simultaneous HDR overexpression and NHEJ suppression using CRISPR-Cas9
Source: Hortic Res. 2022 Jul 22;9:uhac154. doi: 10.1093/hr/uhac154 (PMC9478684; doi:10.1093/hr/uhac154)
Supplement: Web_Material_uhac154 [file web_material_uhac154.zip › Supplementary Figure 7.pptx]

## Slide 1
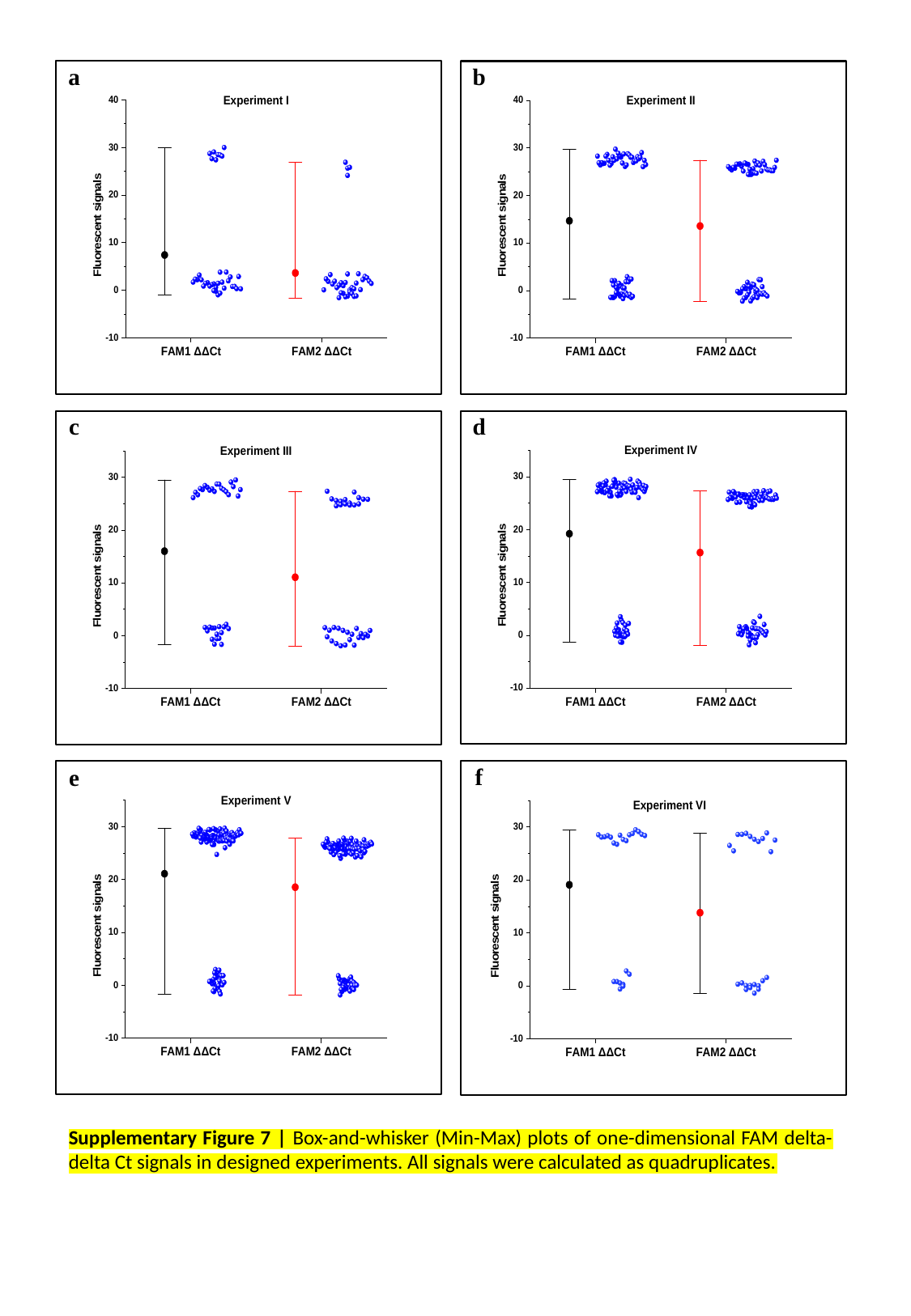

a
b
c
d
f
e
Supplementary Figure 7 | Box-and-whisker (Min-Max) plots of one-dimensional FAM delta-delta Ct signals in designed experiments. All signals were calculated as quadruplicates.
